# Reversible epigenetic alterations mediate PSMA expression heterogeneity in advanced metastatic prostate cancer

**DOI:** 10.1172/jci.insight.162907

**Published:** 2023-04-10

**Authors:** Erolcan Sayar, Radhika A. Patel, Ilsa M. Coleman, Martine P. Roudier, Ailin Zhang, Pallabi Mustafi, Jin-Yih Low, Brian Hanratty, Lisa S. Ang, Vipul Bhatia, Mohamed Adil, Hasim Bakbak, David A. Quigley, Michael T. Schweizer, Jessica E. Hawley, Lori Kollath, Lawrence D. True, Felix Y. Feng, Neil H. Bander, Eva Corey, John K. Lee, Colm Morrissey, Roman Gulati, Peter S. Nelson, Michael C. Haffner

**Affiliations:** 1 Division of Human Biology, Fred Hutchinson Cancer Center, Seattle, Washington, USA.; 2 Department of Urology, University of Washington (UW), Seattle, Washington, USA.; 3 UCSF Helen Diller Family Comprehensive Cancer Center, San Francisco, California, USA.; 4 Division of Medical Oncology, Department of Medicine, UW, Seattle, Washington, USA.; 5 Division of Clinical Research, Fred Hutchinson Cancer Center, Seattle, Washington, USA.; 6 Department of Laboratory Medicine and Pathology, UW, Seattle, Washington, USA.; 7 Department of Urology, Weill Cornell Medicine, New York, New York, USA.; 8 Division of Public Health Sciences, Fred Hutchinson Cancer Center, Seattle, Washington, USA.

**Keywords:** Oncology, Drug therapy, Epigenetics, Prostate cancer

## Abstract

Prostate-specific membrane antigen (PSMA) is an important cell surface target in prostate cancer. There are limited data on the heterogeneity of PSMA tissue expression in metastatic castration-resistant prostate cancer (mCRPC). Furthermore, the mechanisms regulating PSMA expression (encoded by the *FOLH1* gene) are not well understood. Here, we demonstrate that PSMA expression is heterogeneous across different metastatic sites and molecular subtypes of mCRPC. In a rapid autopsy cohort in which multiple metastatic sites per patient were sampled, we found that 13 of 52 (25%) cases had no detectable PSMA and 23 of 52 (44%) cases showed heterogeneous PSMA expression across individual metastases, with 33 (63%) cases harboring at least 1 PSMA-negative site. PSMA-negative tumors displayed distinct transcriptional profiles with expression of druggable targets such as MUC1. Loss of PSMA was associated with epigenetic changes of the *FOLH1* locus, including gain of CpG methylation and loss of histone 3 lysine 27 (H3K27) acetylation. Treatment with histone deacetylase (HDAC) inhibitors reversed this epigenetic repression and restored PSMA expression in vitro and in vivo. Collectively, these data provide insights into the expression patterns and regulation of PSMA in mCRPC and suggest that epigenetic therapies — in particular, HDAC inhibitors — can be used to augment PSMA levels.

## Graphical abstract

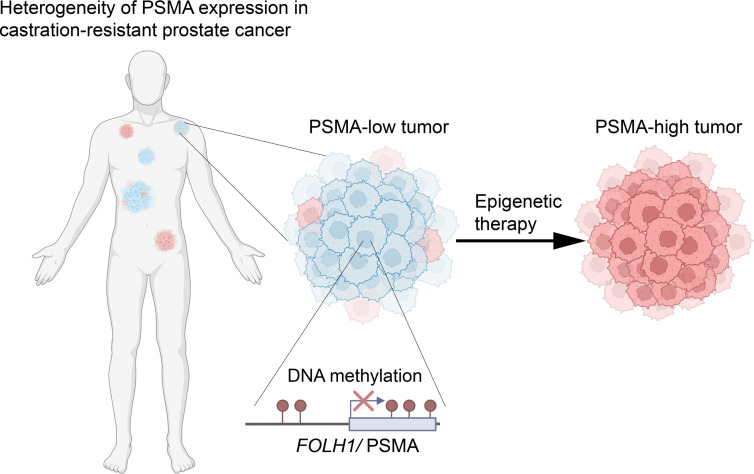

## Introduction

Prostate-specific membrane antigen (PSMA, folate hydrolase I, glutamate carboxypeptidase II) is a type II transmembrane glycoprotein that is expressed in benign prostatic tissue and at higher levels in prostate cancer (1–3). Outside the prostate, PSMA expression is limited to a small number of tissues (1–3). Its large extracellular domain and restricted tissue expression make PSMA a valuable prostate-specific theranostic target (1–3). Several PSMA-targeting therapeutics (including radioligand therapies, antibody-drug conjugates, and cell-based immunotherapies) and PSMA-based imaging modalities have been developed (1, 2). PSMA–positron emission tomography (PET) ligands (^68^Ga-PSMA-11, ^18^F-DCFPyL) have rapidly gained a foothold in clinical practice for tumor staging (4). The recently FDA-approved PSMA-radiopharmaceutical ^177^Lu-PSMA-617 is one of several potentially novel PSMA-targeting therapies that have demonstrated clinical activity in advanced prostate cancer (5, 6).

Despite the overall enthusiasm for PSMA as a target, relatively little is known about the expression and regulation of PSMA in metastatic castration-resistant prostate cancer (mCRPC) (1, 2). Previous studies have shown that PSMA is expressed in the majority of localized prostate cancers (7–9); however, imaging studies of mCRPC have demonstrated that up to 30% of patients have PSMA-negative tumors (3, 10). The small number of prior tissue-based PSMA expression studies in metastatic prostate cancer relied primarily on the evaluation of a single metastatic sample from each patient, which precluded the assessment of the complex expression heterogeneity that may be present across different metastatic sites (11–13).

Furthermore, the transcriptional control of PSMA is poorly understood. Although several transcription factors have been implicated in the regulation of PSMA, the molecular mechanisms that contribute to loss of PSMA expression have not been elucidated (14, 15).

PSMA expression heterogeneity in mCRPC likely poses a critical barrier to the clinical success of PSMA-targeting approaches (1, 12). This was emphasized by recent preclinical and clinical studies demonstrating that the efficacy of ^177^Lu-PSMA-617 is tightly correlated with PSMA levels, and high and homogeneous PSMA expression is required for optimal therapeutic response (16–21). Therefore, it is important to understand the expression patterns of PSMA to determine and anticipate potential resistance mechanisms of PSMA-directed therapies.

Here, we sought to comprehensively assess the inter- and intratumoral heterogeneity of PSMA expression in lethal metastatic prostate cancer. We also aimed to determine the mechanisms governing PSMA expression loss and to investigate the molecular characteristics of PSMA-negative tumors. Collectively, these studies provide potentially novel insights into the expression patterns and transcriptional regulation of PSMA, with important translational implications for PSMA-targeting strategies in mCRPC.

## Results

### PSMA expression differs across molecular subtypes of prostate cancer.

**Figure 1:**
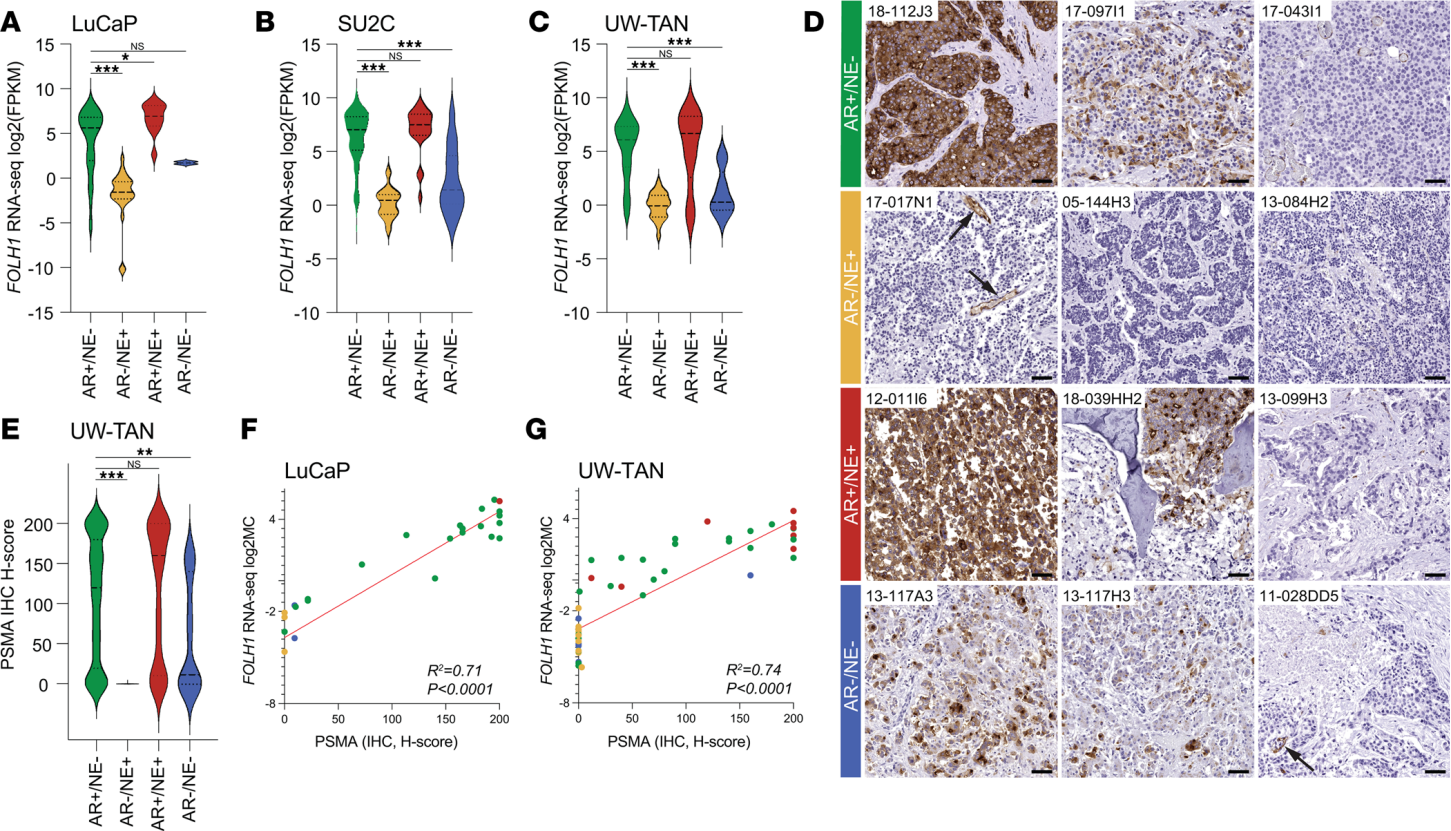
PSMA expression patterns differ between molecular subtypes of lethal metastatic prostate cancer. (**A**–**C**) Violin plots show distribution of *FOLH1* expression (log_2_ FPKM) across molecular subtypes (AR^+^/NE^–^ [green], AR^–^/NE^+^ [yellow], AR^+^/NE^+^ [red], and AR^–^/NE^–^ [blue]), determined by RNA-Seq in LuCaP PDX (*n* = 126) (**A**), SU2C (*n* = 270) (**B**), and UW-TAN (*n* = 172) tumors (**C**) (see Supplemental Table 1 for a breakdown of samples across all molecular phenotypes). (**D**) Representative micrographs of PSMA IHC in different molecular subtypes. Arrows indicate PSMA-positive endothelial cells in cases with absence of tumor cell–specific PSMA expression. (**E**) Violin plot depicts PSMA-expression H-scores from the UW-TAN cohort (*n* = 636). (**F** and **G**) Correlation plots show a significant positive association (*R*^2^ based on Pearson correlation) between PSMA protein expression (by IHC) and *FOLH1* mRNA expression (by RNA-seq) in LuCaP (*n* = 25) (**F**) and UW-TAN samples (*n* = 50) (**G**). Dot colors indicate molecular phenotypes, as above. Scale bars: 50 μm. *P < 0.05; **P < 0.001; ***P < 0.0001, based on Wilcoxon rank tests.

Recent studies have suggested that mCRPC can be divided into 4 molecular subgroups based on androgen receptor (AR) signaling and neuroendocrine (NE) marker expression (22, 23). To determine the expression of PSMA in these clinically relevant subsets, we assessed *FOLH1* mRNA levels in 3 cohorts comprising 126 LuCaP prostate cancer patient-derived xenograft (PDX) samples (Figure 1A), 270 Stand Up To Cancer (SU2C) international dream team mCRPC biopsies (Figure 1B), and 172 lethal metastatic prostate cancer samples from the UW rapid autopsy program (UW Tissue Acquisition Necropsy [UW-TAN]) (Figure 1C, Supplemental Figure 1, and Supplemental Tables 1 and 2; supplemental material available online with this article; https://doi.org/10.1172/jci.insight.162907DS1) (24–26). We observed a high level of expression variability in AR^+^/NE^–^ tumors (mean across cohorts, 48.5 fragments per kilobase of exon per million mapped fragments [FPKM]; range, 0.02–1269). Similar to prior reports, we found low/negative PSMA expression in AR^–^/NE^+^ tumors (NE prostate cancer [NEPC]) (mean across cohorts, 0.59 FPKM; range, 0.0008–8.26) (27). AR^+^/NE^+^ showed expression levels comparable to AR^+^/NE^–^ tumors (mean across cohorts, 84.33 FPKM; range, 0.3–698.4), whereas AR^–^/NE^–^ tumors demonstrated reduced levels (mean across cohorts, 3.40 FPKM; range, 0.3–363.6) (Figure 1, A–C) and AR activity signature scores correlated with PSMA expression (Supplemental Figure 2).

To further determine PSMA protein expression in mCRPC, we performed PSMA IHC on 636 samples from 339 anatomically distinct metastatic sites of 52 cases of the UW-TAN cohort using a previously validated IHC assay (12, 28) (Figure 1D). Protein expression levels differed substantially between AR^+^ and AR^–^ tumors (AR^+^/NE^–^: mean H-score, 106.9; AR^+^/NE^+^: mean H-score, 97.7; AR^–^/NE^+^: mean H-score, 0.06; AR^–^/NE^–^: mean H-score, 50.4) (Figure 1, D and E, Supplemental Figure 3, and Supplemental Table 3). Importantly, while there was a general trend to lower PSMA expression in AR^–^ tumors, we identified several AR^–^/NE^–^ cases with relatively high PSMA H-scores (Figure 1E and Supplemental Figure 4). Similar to the mRNA levels, a significant number of tumors showed no (103 of 339; 30%) or low (H-score ≤ 20) (137 of 339; 40%) PSMA expression. PSMA protein expression levels appeared to follow a bimodal distribution, with the largest groups of samples showing either low or high H-scores. In subset analyses of AR^+^/NE^–^ tumors, we found that 47 of 204 (23%) were low/negative for PSMA. Notably, we observed focal PSMA staining of the tumor-associated vasculature in tumors lacking PSMA expression in tumor cells (Figure 1D and Supplemental Figure 5). To better understand the association between PSMA protein expression (determined by IHC) and *FOLH1* mRNA transcript levels, we analyzed LuCaP PDX and UW-TAN samples with matched IHC and RNA-Seq data (Figure 1, F and G; Supplemental Figure 6; and Supplemental Tables 3 and 4). We observed a strong correlation between protein and mRNA expression, demonstrating that mRNA levels can be used as a proxy for assessing PSMA protein expression.

These data document the diversity of PSMA expression across different molecular subtypes and highlight the high level of overall expression variability in mCRPC.

### PSMA expression shows a high level of inter- and intratumoral heterogeneity.

**Figure 2:**
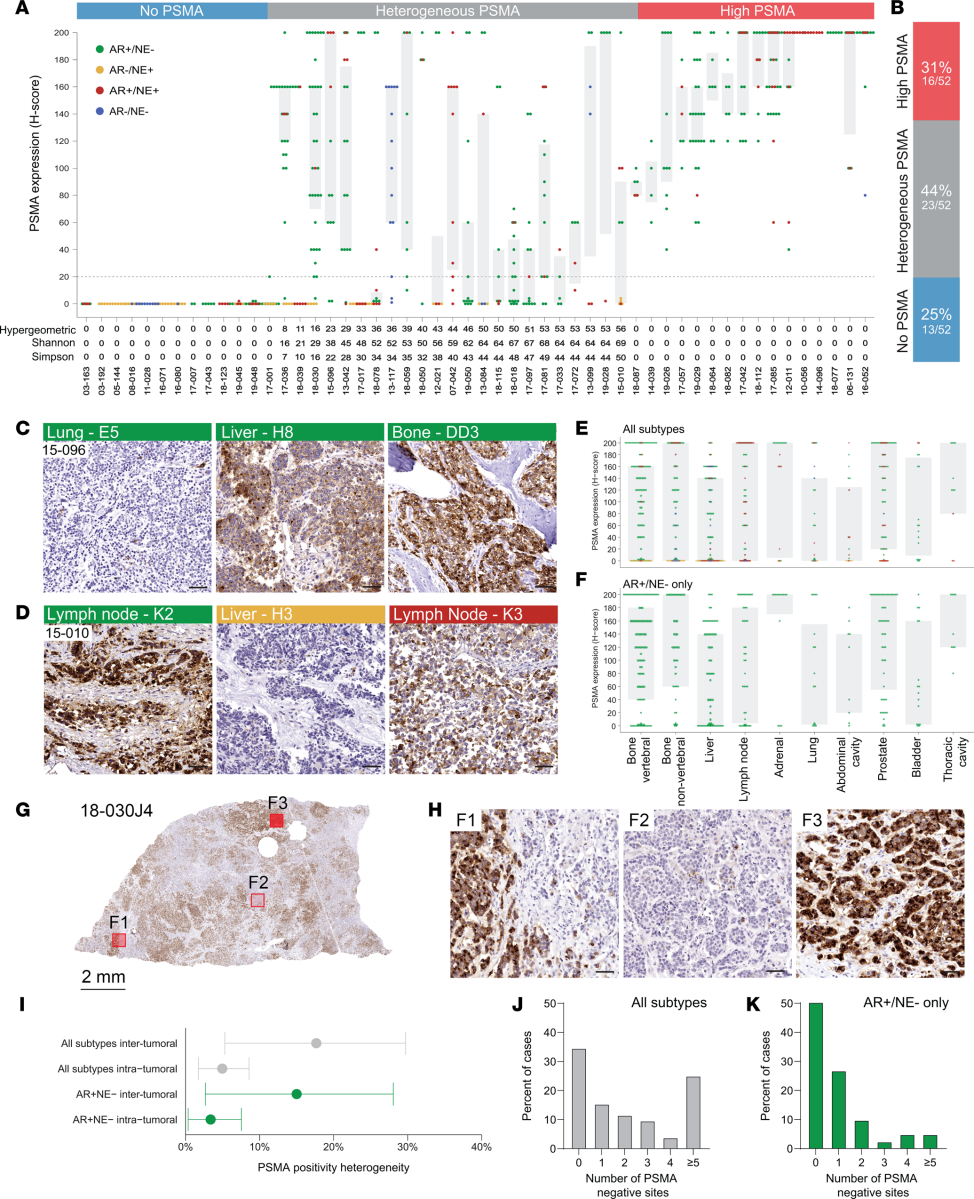
Inter- and intrapatient PSMA expression heterogeneity. (**A**) Dot and box plot show the distribution of PSMA protein expression H-scores in 52 cases from the UW-TAN rapid autopsy cohort (total sample *n* = 636). Each dot represents a tumor sample; the color codes indicate the molecular subtype (AR^+^/NE^–^ [green], AR^–^/NE^+^ [yellow], AR^+^/NE^+^ [red] and AR^–^/NE^–^ [blue]). Gray shadings show interquartile ranges. (**B**) Summary of frequencies of cases with uniformly low/negative PSMA expression (all sites H-score ≤ 20), heterogeneous PSMA expression (both H-scores ≤ 20 and H-score > 20) and uniformly high PSMA expression (all sites with H-scores > 20). (**C**) Representative micrographs of PSMA expression patterns in 3 anatomically distinct metastatic sites (all AR^+^/NE^–^) from 1 case (case 15-096). (**D**) PSMA expression in a case with divergent subtypes (case 15-010). (**E** and **F**) Distribution of PSMA expression H-scores across different organ sites in all tumors (**E**) and AR^+^/NE^–^ tumors (**F**). (**G**) PSMA expression heterogeneity within a metastatic lesion (intratumoral heterogeneity). Left panel shows low power (1***×***) view of a full-face tumor section. Areas with divergent PSMA expression are indicated (F1–F3). (**H**) High-power view (20***×***) of 3 tumor foci (F1–F3 from **G**) showing high level intratumoral expression heterogeneity. (**I**) Mean (95% CI) hypergeometric PSMA expression heterogeneity indices across different metastatic sites in a given patient (intertumoral heterogeneity) and within a metastatic site (intratumoral heterogeneity) for the entire cohort (gray) and AR^+^/NE^–^ tumors (green). (**J** and **K**) Percentage of cases with no (0), 1, 2, 3, 4 or ≥ 5 metastatic sites with a PSMA H-score ≤ 20 (PSMA-negative metastatic sites). Numbers are shown separately for the entire cohort (gray bars) (**J**) and in AR^+^/NE^–^ tumors only (green bars) (**K**). Scale bars: 50 μm.

To further study inter- and intrapatient PSMA expression variability, we leveraged the unique tissue resources and design of the UW-TAN rapid autopsy cohort in which multiple metastatic sites from each patient were sampled. This allowed us to determine both intertumoral (between different metastatic sites) and intratumoral heterogeneity (between different cores from one metastasis). We defined an H-score of ≤ 20 as the cutoff for tumors with low/negative PSMA expression (Supplemental Figure 7). Across the 339 tumors from 52 cases in this cohort, we observed 3 patterns (low/negative, heterogenous, uniformly high) of PSMA expression (Figure 2, A and B). Thirteen of 52 (25%) cases showed low/negative PSMA expression across all metastatic sites (Figure 2, A–D). Although this group was enriched for AR^–^/NE^+^ tumors, it also included several AR^+^/NE^–^ cases. Patients with heterogeneous PSMA expression (defined by the presence of lesions with low/negative PSMA [H-score ≤ 20] and high PSMA [H-score > 20]) composed the largest group (23 of 52; 44%). This group included cases with variable PSMA in metastases of uniform molecular subtype (e.g., case 15-096; Figure 2, A–D, and Supplemental Figures 8 and 9) as well as cases with divergent molecular subtypes in different anatomic sites (e.g., case 15-010; Figure 2, A and D, and Supplemental Figure 8). We observed trends toward lower PSMA H-scores in liver metastases (mean, 66; range, 0–200) and higher PSMA levels in adrenal (mean, 118; range, 0–200) and prostate tumors (mean, 106; range, 0–200) compared with bone sites (vertebral and nonvertebral combined; mean, 88; range, 0–200; *P* = 0.001 and *P* = 0.02, respectively), when including all molecular subtypes (Figure 2E). These trends persisted in subset analyses of AR^+^/NE^–^ tumors (Figure 2F). These findings suggest a potential interplay between the tumor microenvironment of metastatic sites and PSMA expression.

In addition to intertumoral heterogeneity, we also noted substantial intratumoral differences in PSMA expression (Figure 2, G and H; Supplemental Figure 10; and Supplemental Table 3). In full face tissue sections, we identified spatially separate cell clusters with distinct PSMA expression. This demonstrated that PSMA-high and PSMA-negative cell populations exist within a given metastasis (Figure 2, G and H).

To formally quantify the variability in PSMA expression, we examined 3 measures of heterogeneity (hypergeometric probabilities and Shannon and Simpson indices; Figure 2, A and I). To account for PSMA expression heterogeneity driven by molecular subtype–specific expression patterns, we performed additional subset analyses on AR^+^/NE^–^ tumors. We assessed PSMA heterogeneity across different metastatic sites in a patient (intertumoral) and within an individual metastatic site (intratumoral) (Figure 2I). We observed similar levels of PSMA expression heterogeneity in AR^+^/NE^–^ tumors (estimated probability of intertumoral differences, 15% [95% CI, 3%–28%]; estimated probability of intratumoral differences, 3% [95% CI, 0%–8%]) compared with the entire unselected cohort (estimated probability of inter-tumoral differences, 18% [95% CI, 5%–30%]; estimated probability of intratumoral differences, 5% [95% CI, 2%–9%]) (Figure 2I). Next, we determined the frequency of PSMA-low/negative metastases. In unselected patients of all molecular subtypes, only 18 of 52 (34.6%) showed PSMA expression (H-scores > 20) in all metastatic sites (Figure 2J). In contrast, 21 of 41 (51.2%) patients with AR^+^/NE^–^ tumors showed PSMA expression in all metastatic sites, whereas 2 of 41 (4.9%) had 5 or more PSMA-negative sites (Figure 2K). PSMA expression heterogeneity indices were slightly decreased in patients treated prior to the widespread use of abiraterone and enzalutamide (ENZA); however, this trend was not statistically significant (Supplemental Figure 11). These findings suggest that treatment with second-generation AR signaling inhibitors does not profoundly alter PSMA expression patterns.

### PSMA low/negative tumors show distinct transcriptional changes.

**Figure 3:**
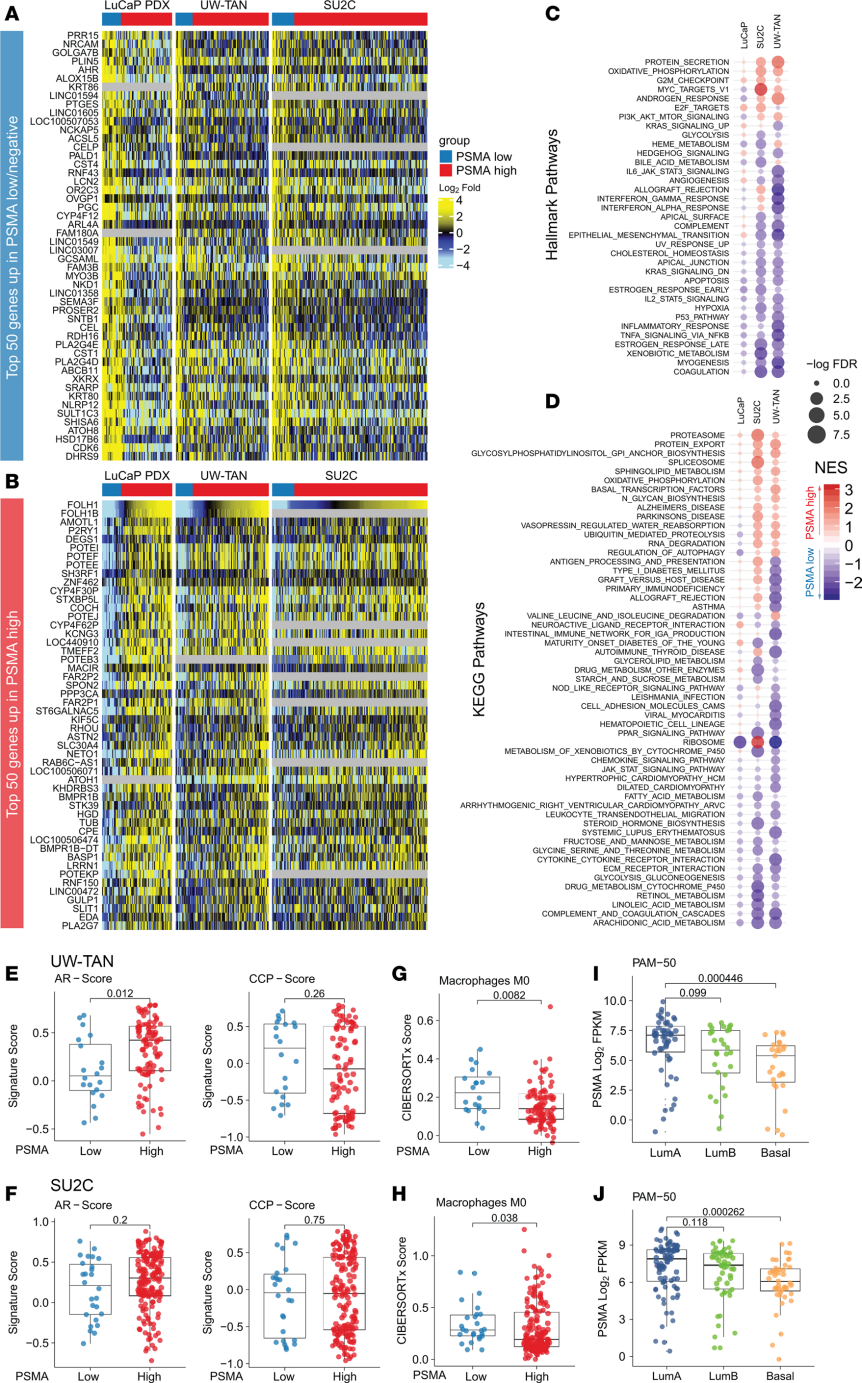
Tumors with low/negative PSMA expression show distinct expression changes. (**A** and **B**) Heatmap showing the top 50 genes upregulated in AR^+^/NE^–^ tumors from the LuCaP PDX (*n* = 82), UW-TAN (*n* = 109), and SU2C (*n* = 182) cohorts with low/negative PSMA expression (**A**) and high PSMA expression (**B**). (**C** and **D**) Gene set enrichment analyses using Hallmark Pathways (**C**) and KEGG Pathways (**D**) show gene sets enriched in PSMA high (red) and PSMA-low/negative (blue) tumors. NES denotes normalized enrichment score. (**E** and **F**) Comparisons of AR signaling activity (AR-score) and cell proliferation (CCP-score) using gene set variation analyses (Supplemental Figures 12 and 13) between PSMA-low/negative and PSMA-high AR^+^/NE^–^ tumors in the UW-TAN (**E**) and SU2C (**F**) cohorts. (**G** and **H**) CIBERSORTx analyses demonstrate differences in macrophage infiltration between PSMA high and PSMA low/negative tumors in UW-TAN (**G**) and SU2C (**H**) cohorts. (**I** and **J**) Differences in PSMA expression based on luminal A, luminal B, and basal PAM-50 status in UW-TAN (**I**) and SU2C (**J**) cohorts. *P* values are based Wilcoxon rank tests.

Next, we aimed to assess transcriptional changes associated with low/negative PSMA/*FOLH1* expression. Since previous reports and our own analyses show differences in PSMA expression across different molecular subtypes (Figure 1), with the lowest levels observed in AR^–^/NE^+^ tumors, we restricted our analyses to AR^+^/NE^–^ tumors to avoid any confounding effects by subtype-specific expression patterns. We first performed differential gene expression analyses using previously published RNA-Seq data of 82 LuCaP PDX, 109 UW-TAN, and 182 SU2C AR^+^/NE^–^ tumors (24–26). Comparing tumors with high PSMA expression to PSMA-low/negative tumors, we identified 104 genes with higher expression in PSMA-low/negative tumors and 44 genes with higher expression in PSMA-high tumors in at least 2 of 3 cohorts with a FDR < 0.05 (Figure 3, A and B; Supplemental Figure 12; and Supplemental Tables 5 and 6). Gene set enrichment analyses (GSEA) revealed increased activity of inflammatory response, hypoxia, epithelial mesenchymal transition, and metabolic and glycolytic pathways in PSMA-low/negative tumors (Figure 3, C and D). Similar results were observed when tumors of all molecular subclasses were analyzed, but a strong enrichment of genes involved in cell proliferation was noted in PSMA-low tumors, likely due to the overrepresentation of AR^–^/NE^+^ tumors in the PSMA-low/negative group (Supplemental Figures 13 and 14). The PSMA-associated expression signatures (Figure 3, A and B) showed differential enrichment in AR^+^ and AR^–^ tumors (Supplemental Figure 15), suggesting an association between this signature and molecular subtypes.

To further characterize clinically relevant molecular features of PSMA-low/negative AR^+^/NE^–^ tumors, we determined the association between PSMA expression and AR activity (23) and Cell Cycle Progression (CCP) scores (29) in AR^+^/NE^–^ tumors using gene set variation analysis (GSVA) (23). We observed a trend toward lower AR activity in PSMA-low/negative tumors, which was statistically significant only in the UW-TAN cohort (*P* =0.012); we observed no statistically significant differences in CCP scores (Figure 3, E and F). Given the differences in genes involved in immune signaling observed in GSEA, we used CIBERSORTx analyses to deconvolute immune infiltrates and found a significant increase in M0 macrophages in PSMA-low/negative tumors in the SU2C and UW-TAN cohorts (Figure 3, G and H) (30). Although additional differences in immune cell composition were noted, none reached statistical significance in both data sets (Supplemental Figures 16 and 17). Applying the PAM-50 classifier, which allows for the classification of tumors based on a 50-gene signature into luminal A, luminal B, and basal subtypes, we observed an enrichment of lower PSMA expression in tumors of the basal subtype (Figure 3, I and J) (31).

### Targetable alterations in PSMA-low/negative tumors.

**Figure 4:**
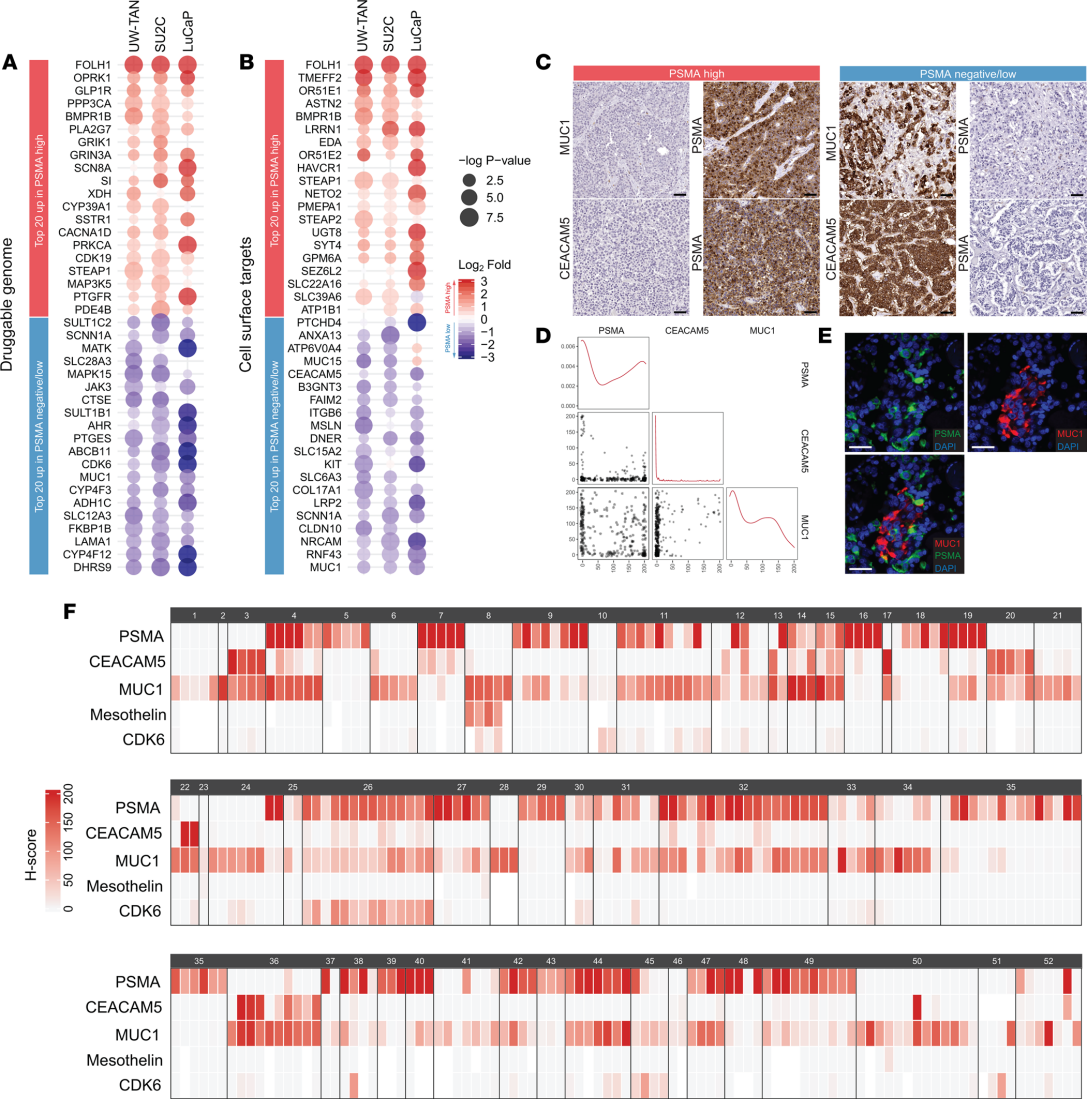
PSMA-low/negative tumors show targetable alterations. (**A**) Heatmap of top 20 differentially expressed genes with annotated drug target properties from the druggable genome database (rank ordered based on fold expression difference) between PSMA-high (red) and PSMA-low/negative (blue) AR^+^/NE^–^ tumors in UW-TAN, SU2C, and LuCaP PDX cohorts. (**B**) Top 20 differentially expressed genes encoding for cell surface proteins between PSMA-high (red) and PSMA-low/negative (blue) tumors in UW-TAN, SU2C, and LuCaP PDX. Heatmaps are sorted by rank order based on mean fold change differences, and directionality is color coded: red, higher in PSMA-high; blue, higher in PSMA-low/negative. (**C**) Representative micrographs of CEACAM5 and MUC1 IHC in PSMA-negative/low and PSMA-high tumors. (**D**) Correlation plots for PSMA, MUC1, and CEACAM5 protein expression. (**E**) Dual fluorescence images showing distinct cell population labeling for MUC1 (red) and PSMA (green). (**F**) Heatmaps of PSMA, CEACAM5, MUC1, mesothelin, and CDK6 expression based on IHC H-scores across 289 metastatic sites in 52 patients. Expression scores for each protein target are color coded from light gray to red. White boxes indicate missing data. Each colored box represents a metastatic site; black boxes outline each case (see Supplemental Table 8 for UW-TAN case identifiers). Scale bars: 50 μm.

Next, we sought to investigate whether PSMA-low/negative tumors showed shared targetable alterations. To this end, we used the druggable genome database, a compendium of putative and validated drug targets (32). We determined the top 20 differentially expressed drug targets, which revealed dehydrogenase/reductase family member 9 (DHRS9), Janus kinase 3 (JAK3), megakaryocyte-associated tyrosine kinase (MATK), prostaglandin E synthase (PTGES), alcohol dehydrogenase 1C (ADH1C), mitogen-activated protein kinase 15 (MAPK15), and cyclin-dependent kinase 6 (CDK6) as consistently upregulated in PSMA-low/negative tumors across all cohorts (Figure 4A and Supplemental Tables 5–7). Given the role of PSMA as a target for antibody-based and cellular therapies, we further assessed the expression differences of 409 cell surface proteins previously described in the Cancer Surfaceome Atlas (33). We observed consistently increased expression of several validated cell surface targets on the mRNA level, including mucin 1 (MUC1, also known as epithelial membrane antigen [EMA]), mesothelin (MSLN), and carcinoembryonic antigen-related cell adhesion molecule 5 (CEACAM5) in PSMA-low/negative tumors (Figure 4B) (34–36).

To corroborate these in silico findings, we performed IHC studies for CEACAM5, MUC1, MSLN, and CDK6 in an additional cohort of 52 rapid autopsy cases (Figure 4, C–F). While the protein expression of MSLN and CDK6 was restricted to a small number of cases, MUC1 and CEACAM5 were expressed in a large fraction of metastases (Figure 4, D and F). Importantly, out of 102 tumors with low/no PSMA expression (H-score < 20), 91 (89.2%) showed MUC1 expression (mean H-score = 92). Conversely, in the 88 tumors with low/no MUC1 expression, PSMA was expressed in 73 samples (83%) (Figure 4F). These findings suggest inverse expression of PSMA and MUC1. In support of this notion, dual immunofluorescence labeling studies in PSMA- and MUC1-positive cases demonstrated that MUC1 and PSMA were expressed in separate cell populations with limited coexpression at the single-cell level (Figure 4E). Collectively, these findings indicate that tumors with low/negative PSMA expression exhibit distinct transcriptional changes. Furthermore, we show that MUC1 represents a relevant surface target in PSMA-low/negative tumors.

### AR signaling can modulate PSMA expression.

**Figure 5:**
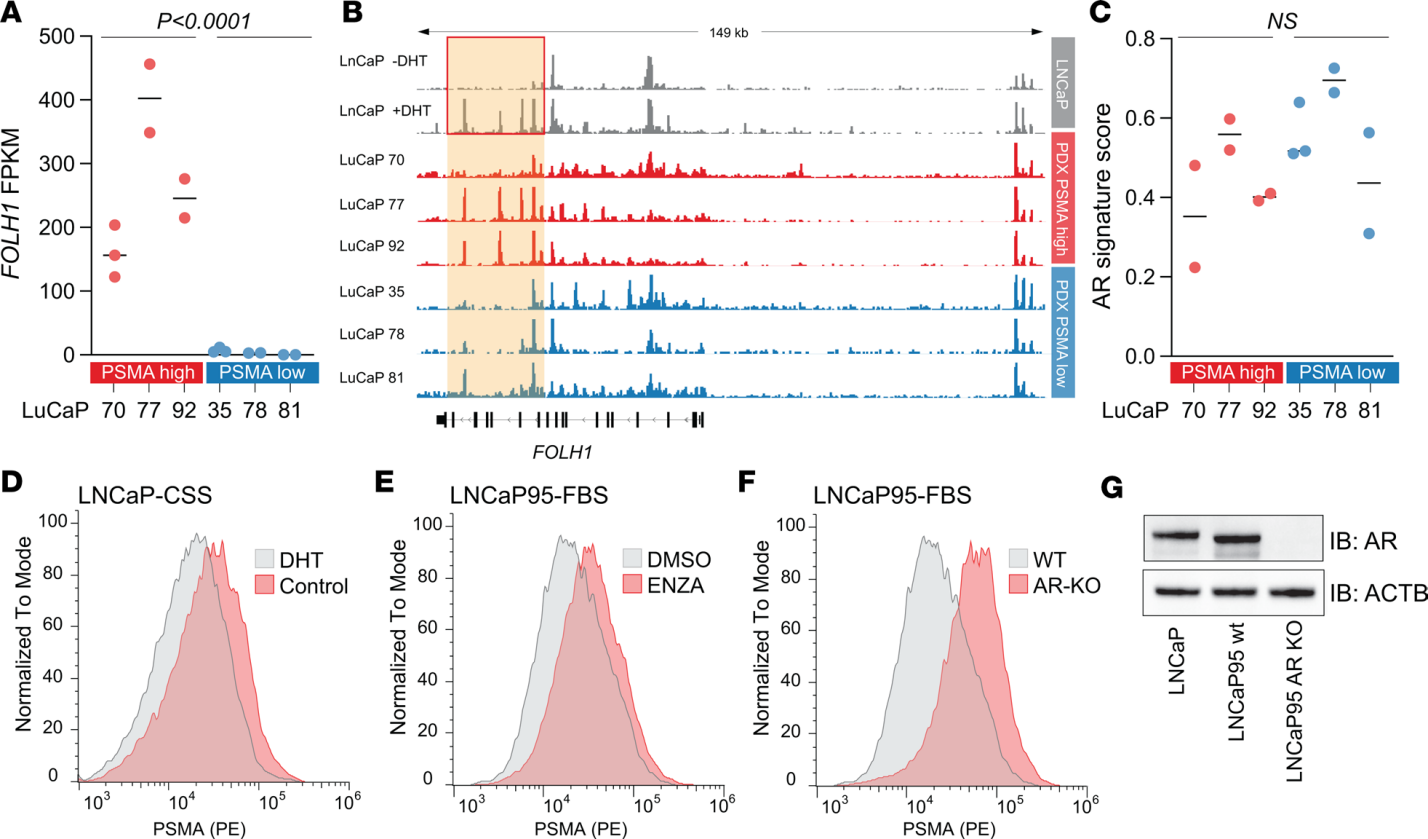
AR-mediated changes in PSMA expression. (**A**) *FOLH1* mRNA expression in AR^+^ LuCaP PDX lines. (**B**) AR ChIP-Seq tracks in LNCaP cell line and LuCaP PDX tissues show AR recruitment at the *FOLH1* locus. Red box highlights dihydrotestosterone-induced (DHT-induced) peaks in LNCaP cells. (**C**) Distribution of 10-gene AR signature (23) across LuCaP models. *P* values are based on 2-tailed *t* tests. (**D** and **E**) Density plots show PSMA cell surface expression in LnCaP and LNCaP95 (as indicated) in the absence or presence of 10 nM DHT (**D**) or 10μM enzalutamide (ENZA) (**E**) for 6 days. (**F**) PSMA cell surface expression in LNCaP95 AR-KO and parental WT cells. (**G**) Western blot shows complete loss of AR protein expression in AR-KO cells (40).

Prior studies have suggested that AR signaling can repress PSMA expression (15, 37). This contrasts with our observation in clinical specimens, where loss of AR (in particular in the context of AR^–^/NE^+^ tumors) was associated with reduced/absent PSMA expression (Figure 1 and Supplemental Figure 2). To formally investigate the interaction between AR signaling and PSMA expression, we first determined AR binding patterns at the *FOLH1* gene locus in LNCaP cells (PSMA-high) and 6 LuCaP PDX lines with high (LuCaP 70, 77, and 92) and low (LuCaP 35, 78, and 81) PSMA expression (Figure 5A) using publicly available ChIP-Seq data sets (38). We observed increased androgen induced AR binding at the 3′ end of the *FOLH1* gene (introns 14 and 18) (Figure 5B) in LNCaP cells. Two of the PSMA-high LuCaP lines (LuCaP 77 and 92) showed overlapping peaks at these sites of androgen-induced AR binding, whereas only 1 of the PSMA-low lines (LuCaP 81) showed enrichment coinciding with the peak in intron 18. Notably, AR signaling activity scores were similar between PSMA-high and -low LuCaP lines, but there was a trend toward lower AR mRNA expression in PSMA-low LuCaPs (Figure 5C and Supplemental Figure 18A). These findings show that AR binding patterns or AR signaling activity alone cannot account for differences in PSMA expression.

To mechanistically probe the role of AR signaling in regulating PSMA expression, we assessed PSMA expression by flow cytometry in AR/PSMA-positive LNCaP and LNCaP95 cells. As shown in prior studies, we found that stimulation with dihydrotestosterone (DHT) reduced PSMA expression (Figure 5D and Supplemental Figure 18B), whereas treatment with the competitive AR inhibitor ENZA or androgen deprivation increased PSMA levels (Figure 5E and Supplemental Figure 18C) (15, 37). Similarly, long-term androgen deprivation in 2 independently derived androgen-independent, AR^+^ cell line models also resulted in increased PSMA levels (Supplemental Figure 18D) (39). Since we observed low levels of PSMA in AR^–^ tumors, we further aimed to determine PSMA expression in the context of AR depletion in AR-KO cells as well as in cells treated with the AR degrader ARCC-4 (40, 41). Both long-term genetic (LNCaP95 AR-KO; Figure 5, F and G) and short-term pharmacologic depletion of AR resulted in increased PSMA expression (Supplemental Figure 18, E and F). In summary, although AR signaling can modulate PSMA expression, our data demonstrate that AR loss and low AR activity are likely insufficient to reduce PSMA expression in cell line models. Therefore, alternative epigenetic mechanisms likely regulate *FOLH1* expression.

### Cooperating epigenetic changes associate with PSMA silencing.

**Figure 6:**
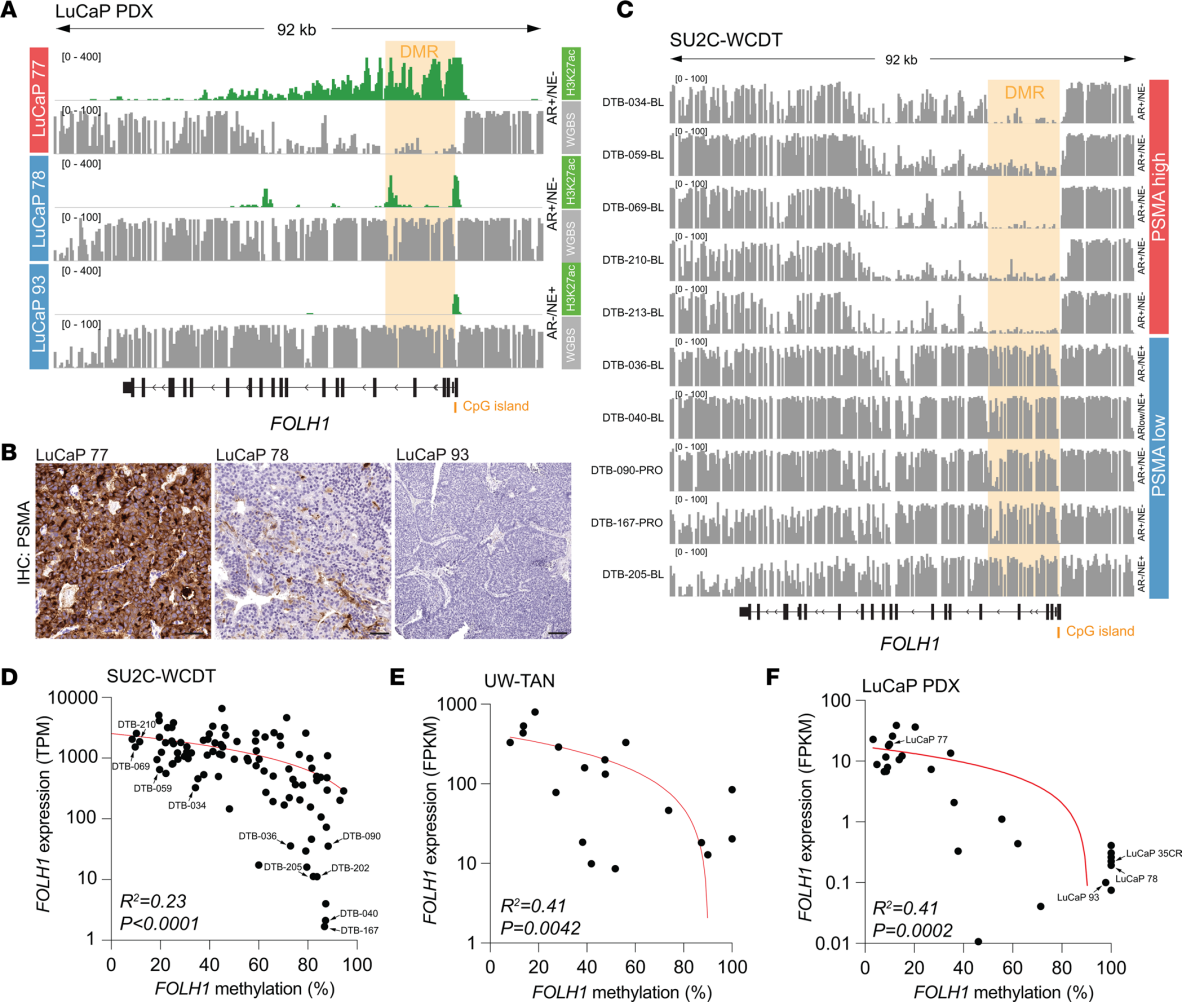
Epigenetic changes enforce silencing of FOLH1/PSMA. (**A**) Whole-genome bisulfite sequencing (WGBS) tracks from PSMA-high (LuCaP 77, red) and PSMA-negative (LuCaP 78 and 93, blue) tumors reveal a differentially methylated region (DMR) encompassing the first 14 kb of the *FOLH1* gene and inverse enrichment for H3K27ac. (**B**) Micrographs of PSMA IHC of LuCaP 77, LuCaP 78, and LuCaP 93 demonstrate the difference in PSMA expression. (**C**) Representative WGBS tracks of mCRPC tumors from the SU2C-WCDT cohort show gain of methylation in the DMR in PSMA-low/negative tumors. (**D**–**F**) Scatter plots show the correlation between *FOLH1* expression (based on RNA-Seq) and DMR methylation derived from WGBS (SU2C-WCDT, *n* = 98) (**D**) and targeted COMPARE-MS analyses UW-TAN (*n* = 18) (**E**) and LuCaP (*n* = 29) (**F**) cohorts. Curves were fit by linear regression, and *R*^2^ and *P* values were derived by Pearson correlation.

The variability in PSMA expression prompted us to further determine independent of AR mechanisms of PSMA regulation. To this end, we first assessed epigenetic features of the *FOLH1* gene locus in LuCaP PDX lines with distinct PSMA protein expression (Figure 6, A and B). Using whole-genome bisulfite sequencing (WGBS), we identified an approximately 14 kb region of the *FOLH1* gene encompassing the first 5 introns, but not a CpG island at the transcriptional start site, which showed CpG hypermethylation in the PSMA-negative PDX lines LuCaP 78 and 93 (Figure 6A and Supplemental Figure 19). In PSMA expressing LuCaP 77, this differentially methylated region (DMR) was hypomethylated. Furthermore, leveraging ChIP-seq and ATAC-Seq chromatin profiles of LuCaP PDX lines (42, 43), we observed that DNA methylation was inversely correlated with chromatin accessibility and enrichment for histone 3 lysine 27 acetylation (H3K27ac) (Figure 6A and Supplemental Figures 20 and 21).

To investigate the association between DNA methylation and *FOLH1* expression in clinical specimens, we analyzed a previously published series of CRPC WGBS samples (SU2C-WCDT, *n* = 98) (Figure 6C) (44). Similar to LuCaP PDX models, we observed a robust difference in *FOLH1* gene methylation profiles between PSMA-high and PSMA-low tumors (Figure 6, C and D). The tight correlation between PSMA/*FOLH1* expression and *FOLH1* CpG methylation observed in WGBS studies was further corroborated using a targeted methylation enrichment assay (COMPARE-MS) in additional LuCaP PDX (*n* = 29) and UW-TAN rapid autopsy tissue samples (*n* = 18) (Figure 6, E and F). Of note, *FOLH1* hypermethylation was observed in both AR^+^ and AR^–^ tumors, and all AR^–^/NE^+^ samples showed CpG hypermethylation (Figure 6, A and C). These findings demonstrate that, independently of AR or NE status, DNA methylation changes are tightly linked to PSMA expression and that *FOLH1* gene regulation is controlled by an interplay of repressive (DNA hypermethylation) and active (H3K27ac) chromatin modifications.

### Epigenetic therapies can restore and augment PSMA expression.

**Figure 7:**
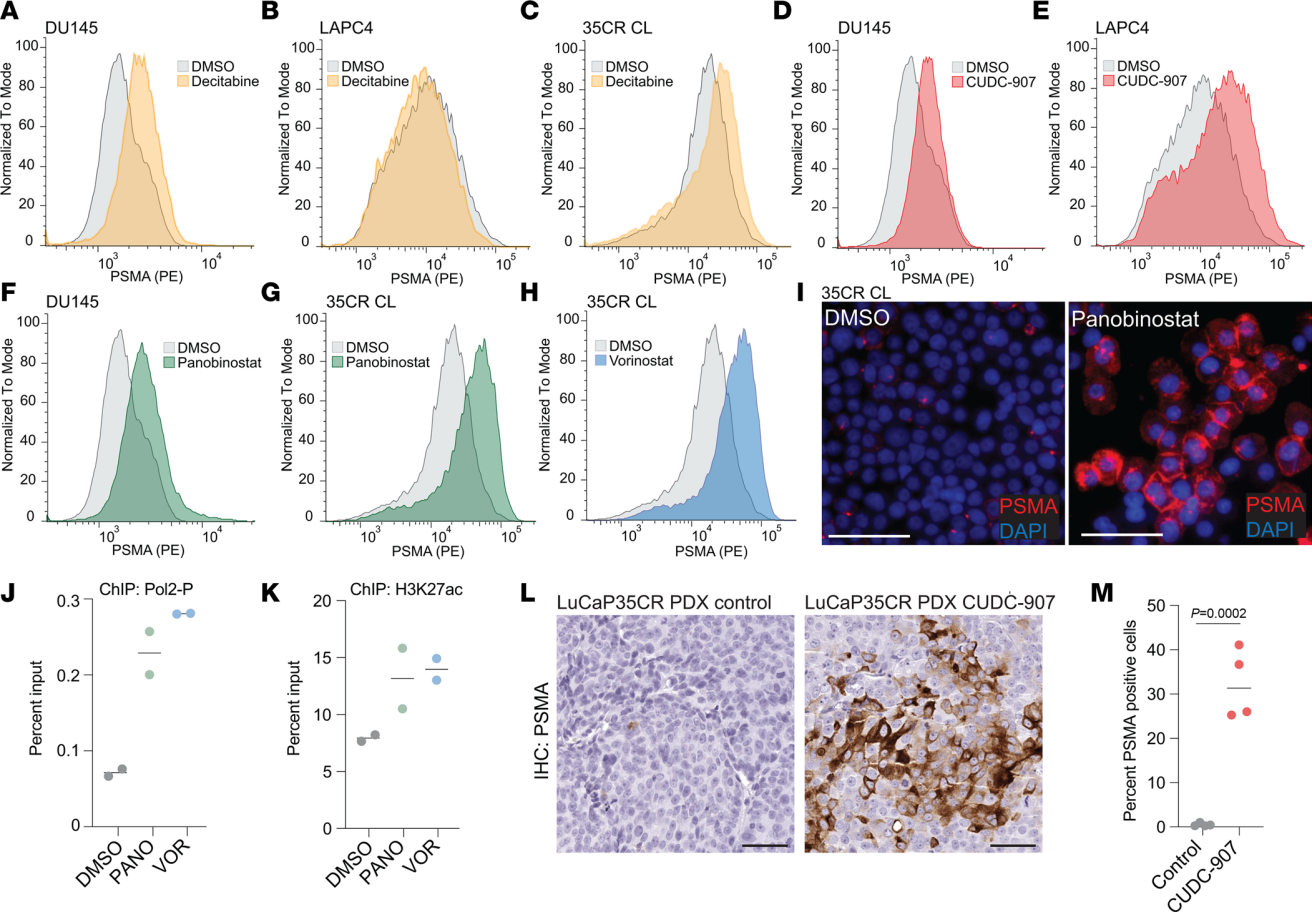
Pharmacologic epigenetic modifiers reverse PSMA silencing. (**A**–**C**) Density plots show PSMA cell surface expression in DU145 (**A**), LAPC4 (**B**), and LuCaP 35CR cell line (CL) (**C**) cells treated for 6 days with vehicle control (DMSO) or 500 nM decitabine (DAC). (**D**–**H**) PSMA expression in DU145, LAPC4, and LuCaP 35CR CL cells treated for 6 days with panobinostat (PANO, 10 nM), CUDC-907 (50 nM), or vorinostat (VOR, 1 μM). (**I**) Representative micrographs of cytospin preparations of LuCaP 35CR CL treated with DMSO or panobinostat (10 nM) stained for PSMA (red) and DAPI (blue). (**J** and **K**) Chromatin immunoprecipitation studies show serine 5 phosphorylated RNA polymerase 2 (Pol2-P) (**J**) and H3K27ac (**K**) enrichment normalized to input in LuCaP 35CR CL treated with vorinostat (VOR, 1 μM), panobinostat (PANO, 10 nM), or DMSO. (**L**) Micrographs of LuCaP 35CR PDX tumors stained for PSMA grown in mice treated with solvent control (30% captisol) or CUDC-907 at a dose of 75 mg/kg/day for 21 days. (**M**) Percent of PSMA-positive cells in control and CUDC-907 treated tumors (*n* = 4 per group). *P* value are based on 2-tailed *t* tests. Scale bars: 50 μm.

Epigenetic changes are known to be at least partly reversible. Pharmacologic inhibitors of enzymes involved in DNA methylation (DNA methyltransferases [DNMT]) and histone acetylation (histone deacetylases [HDAC]) have been developed and explored clinically to modulate epigenetic states in cancers (45). Given our insights into the epigenetic regulation of PSMA that involves the interplay between DNA methylation and histone acetylation, we aimed to test if DNMT inhibitors (DNMTi) or HDAC inhibitors (HDACi) could augment PSMA expression in cell line models (Figure 7). To this end, we treated 3 cell line models (DU145, LAPC4, and LuCaP 35CR CL) with the DNMTi decitabine and pan-HDACi (panobinostat, CUDC-907, vorinostat) and applied 2 orthogonal approaches (flow cytometry and fluorescence immunocytochemistry) and 2 separate monoclonal antibodies (3E6 and LNI-17) to measure PSMA protein expression. Decitabine treatment alone resulted in increased expression in DU145 and LuCaP 35CR CL cells (Figure 7, A–C, and Supplemental Figures 22–24), suggesting that DNMTi is effective in enhancing PSMA expression. HDACi treatment, however, increased PSMA protein expression in all lines, irrespective of baseline PSMA levels (Figure 7, D–I). A modest additive effect was noted when decitabine was combined with HDACi in LAPC4 and LuCaP 35CR CL cells (Supplemental Figures 22 and 23). To assess epigenetic changes associated with HDACi and DNMTi treatment, we performed targeted ChIP experiments for H3K27ac and serine 5 phosphorylated RNA polymerase 2 (Pol2-P), the transcriptionally active form of the enzyme. We observed an increase in both Pol2-P and H3K27ac at the *FOLH1* locus upon HDACi and DNMTi treatment in LAPC4 and LuCaP 35CR CL cells (Figure 7, J and K, and Supplemental Figure 25), consistent with an actively transcribed chromatin state.

Lastly, to determine whether these in vitro results would also extend to in vivo responses, we treated mice bearing LuCaP 35CR xenografts (a PDX line in which the *FOLH1* locus is methylated, which shows very low/no baseline PSMA expression; Figure 6F) with the pan-HDACi CUDC-907. Tumors harvested after 3 weeks of treatment with CUDC-907 showed significantly increased PSMA expression, whereas only very rare positive cells were detected in control-treated animals (Figure 7, L and M).

These data suggest that the epigenetic changes repressing PSMA transcription are potentially reversible and that epigenetic drugs — in particular, HDACi — can be used to augment PSMA expression.

## Discussion

Targeting cell surface proteins for therapy and diagnostics has become an important cornerstone in solid tumor oncology (33). PSMA is the most extensively validated cell surface target in prostate cancer (1–3). PSMA-directed therapeutics have shown highly encouraging clinical activity, but a significant percentage of patients does not respond (1, 2, 5, 6). Not unexpectedly, several recent analyses have shown that the level of PSMA expression is correlated with therapeutic response rates to ^177^Lu-PSMA-617, suggesting that target expression is a key determinant for the success of PSMA-directed therapies (16–21). More broadly, optimal patient selection, assessment of resistance mechanisms, and cotargeting strategies will be important to enhance the clinical benefit of PSMA-targeting agents in the future.

Prior reports have assessed the expression of PSMA in metastatic prostate cancer tissues using IHC and showed absence of PSMA expression in 16%–27% of cases (11–13). One limitation of these previous tissue-based PSMA expression studies is that they relied mostly on single-lesion sampling and, therefore, did not provide information on the heterogeneity of PSMA expression between different metastatic sites. To address this issue, we determined the expression of PSMA in a cohort of 52 men with lethal metastatic prostate cancer who underwent a rapid autopsy, and metastatic tissues representative of the entire metastatic tumor burden were procured. This unique cohort allowed us to comprehensively assess PSMA expression across different metastatic sites in a large number of patients. While we observed PSMA positivity across all metastatic sites in 31% of cases, the vast majority of patients (69%) demonstrated either heterogeneous PSMA expression across different metastatic sites or a complete absence of PSMA labeling. Although we noted that AR^–^/NE^+^ tumors consistently lacked PSMA protein expression, we determined that a substantial number of AR^+^/NE^–^ tumors also showed low levels or absence of PSMA staining. These findings have implications for the clinical implementation of PSMA-targeted therapies and highlight the importance of patient selection (5, 6). Since lesions with low/negative PSMA expression are likely to contribute to resistance to PSMA-targeting agents, standardized imaging- and tissue-based protocols are needed to screen patients prior to therapy.

In our cohort, several tumors that showed no PSMA in tumor cells were positive for neovascular PSMA expression. Although PSMA labeling in tumor-associated vessels has been previously described in other tumor types, the neovasculature of prostate cancer was thought to be less commonly positive for PSMA (46). The enrichment of PSMA-positive vessels in PSMA-negative tumors raises the possibility of developing vascular targeting strategies. In addition, the neovascular expression of PSMA could account for some of the discrepancies observed between PSMA-PET–based and tissue-based studies (46). Similar to previous reports, we observed substantial differences in PSMA expression in different cancer cell clusters within metastatic deposits, demonstrating the high level of intratumoral PSMA expression heterogeneity in mCRPC (12). Collectively, these findings highlight that in situ assessment of PSMA expression can provide information on the localization and distribution of PSMA expression and emphasize the need to correlate and integrate PSMA-PET imaging with tissue-based analyses in future studies (47).

Given the relatively high rate of patients with PSMA-negative metastases, we aimed to further characterize the molecular features of these tumors. Using 3 independent cohorts (LuCaP PDX, SU2C mCRPC biopsies, and UW-TAN autopsy) and focusing specifically on AR^+^/NE^–^ tumors, we found that PSMA-low/negative tumors showed substantially different transcriptomic changes compared with PSMA-expressing tumors. For instance, we noted increased expression in metabolic gene sets, including genes involved in glycolysis, in AR^+^/NE^–^ PSMA-low/negative tumors. These findings potentially explain observations in prior PET imaging studies, indicating that PSMA-negative tumors can have distinct metabolic activity (48). Furthermore, we found an enrichment in inflammatory response and cytokine signaling genes in PSMA-low/negative tumors that was also associated with differences in the composition of the tumor-associated immune microenvironment, with an increase in macrophage infiltration. These observations suggest that PSMA-positive and PSMA-negative tumors broadly exhibit different biological features.

Importantly, several of these biological differences between PSMA-positive and PSMA-negative tumors may be clinically actionable. Our analyses revealed additional targets in PSMA-negative tumors — including CEACAM5, MUC1, and MSLN — for which targeting strategies have already been developed (34–36). In particular, our in silico and in situ studies demonstrate that MUC1 (EMA) could be an attractive target in PSMA-negative tumors, since on the patient, metastasis, and individual tumor cell levels, we observed an inverse correlation between MUC1 and PSMA expression. Of note, MUC1 has been the focus of chimeric antigen receptor T cell (CAR-T) as well as antibody-drug conjugate development efforts for other tumor types (49, 50). Collectively, the insights presented here pave the way for novel tailored strategies for PSMA-negative tumors using combinatorial approaches that target both PSMA-positive and PSMA-negative cell populations.

In addition to targeting unique alterations in PSMA-negative tumors, understanding the mediators of PSMA silencing would enable the development of strategies to enhance PSMA expression and, thus, augment PSMA-targeting therapies. We therefore sought to explore the mechanisms underlying transcriptional silencing of PSMA.

In clinical specimens, we observed that AR-negative tumors tended to have low/absent PSMA levels, suggesting that AR signaling might be required for FOLH1/PSMA expression. This observation contrasts with prior studies demonstrating that, in different model systems, PSMA expression is negatively regulated by AR (15, 51). Here we show that, in AR^+^ tumors, AR binding at the *FOLH1* locus and AR signaling activity did not significantly differ between PSMA-high and PSMA-low/negative samples. Furthermore, genetic and pharmacologic depletion of AR resulted in a modest increase (not decrease) of PSMA expression. Collectively, these data demonstrate that, although AR can modulate *FOLH1*/PSMA expression, loss of AR or reduced AR signaling — at least in prostate cancer model systems — does not result in *FOLH1* silencing; the data therefore suggest that other mechanisms are likely responsible for the profound changes in PSMA expression observed in CRPC.

To explore alternative modes of PSMA silencing, we determined the epigenetic features of the *FOLH1* locus in PSMA-high and PSMA-low/negative models. We observed that tumors with high PSMA expression exhibited changes in both DNA methylation and histone acetylation. Previous reports have suggested potential epigenetic regulation of PSMA by CpG methylation (52, 53). Consistent with an earlier study by Zhao et al., we found that the *FOLH1* locus showed gain of CpG methylation in a subset of CRPC cases (44). However, distinct from the more focal gain of methylation observed in other gene loci, we observed differential methylation of about 14 kb of the *FOLH1* locus outside of a CpG island, which was tightly associated with loss of PSMA expression. Our findings suggest that epigenetic silencing by CpG methylation contributes to PSMA repression in both PSMA-negative adenocarcinomas and NEPCs. The tight inverse association between CpG hypomethylation and H3K27ac, which is a marker of active enhancers and sites of transcription in PSMA-high tumors, reflects the coordinated interplay between DNA and histone modification (54, 55).

Notably, none of the cases studied here has undergone PSMA-directed therapies. Therefore, *FOLH1* hypermethylation likely represents a potential intrinsic resistance mechanism. It remains to be explored whether epigenetic silencing of PSMA is also a relevant resistance mechanism that arises as a consequence of PSMA-directed therapies.

Since epigenetic changes are potentially reversible, we tested different pharmacological epigenetic modifiers to increase PSMA. Previous studies have demonstrated that HDAC inhibition and demethylating agents can act in concert to induce the expression of silenced genes (56, 57). We found that treatment with HDACi (vorinostat, panobinostat, and CUDC-907) resulted in significant upregulation of PSMA expression in vitro and in vivo. While the most consistent reexpression of PSMA was observed with HDACi, DNMT inhibition also showed a modest increase in expression. In addition, we observed differences in the reexpression activity of different HDACi in different cell line models and contexts. While our study provides proof of concept that epigenetic modifiers can be used to augment PSMA expression, future studies are needed to more specifically assess the activity of different HDACi in a broader range of PDX models.

It is intriguing to speculate that a combination of HDACi and PSMA-targeting agents could enhance therapeutic efficacy and mitigate primary or secondary resistance due to epigenetic silencing of PSMA. In this context, it is important to note that prior studies have demonstrated a radio-sensitizing effect of HDACi (58). Therefore, combining HDACi and PSMA radiopharmaceuticals — in particular, ^177^Lu-PSMA-617 — could augment the radiation-induced therapeutic benefit in addition to inducing increased target expression.

This study has several limitations. First, our detailed in situ profiling efforts focus on a cohort of men with lethal metastatic prostate cancer, a disease stage that is known to be characterized by a higher rate of tumoral heterogeneity. It is unclear whether earlier stages of progression also have high levels of inter- and intratumoral heterogeneity. Additionally, the data collected in this study rely on TMAs, which — owing to the more restricted sampling size — may lead to an underestimation of the rate of intratumoral expression heterogeneity. Furthermore, although we used a previously extensively validated antibody for IHC, as with all studies using formalin-fixed paraffin-embedded tissues, it is possible that preanalytical variables could result in a higher rate of false-negative cases. Therefore, additional studies combining both tissue-based and PET imaging techniques are required to determine the true rate of PSMA-negative tumors in large contemporary patient cohorts.

In summary, we showed that PSMA expression is heterogeneous in mCRPC. We found distinct targetable alterations in PSMA-negative tumors and show that PSMA expression can be restored by treatment with epigenetic modifiers. Collectively, these data provide insights into the biology of PSMA and suggest cotargeting approaches to enhance the efficacy of PSMA-targeting therapies in patients with advanced metastatic prostate cancer.

## Methods

### Cell lines and in vitro experiments.

Human prostate cancer cell lines LNCaP and DU-145 were obtained from the American Type Culture Collection (ATCC). LAPC4, LNCaP95, and LNCaP95 AR-KO cells were gifts from John Isaacs (Johns Hopkins School of Medicine, Baltimore, Maryland, USA). The LuCaP 35CR cell line (35CR CL) was derived from the LuCaP 35CR PDX model and provided by Peter Nelson (Fred Hutchinson Cancer Center) (59). All cells were grown in the recommended media supplemented with 10% FBS (Sigma-Aldrich) and maintained at 37°C with 5% CO_2_. Short tandem repeat genotyping was used to authenticate all lines, and cells were confirmed to be mycoplasma free using the MycoAlert Detection Kit (Lonza, LT07-418). Cells were cultured for no more than 10 passages after thawing and before experimental use. ENZA, decitabine, vorinostat, panobinostat, and CUDC-907 were purchased from SelleckChem; ARCC-4 was purchased from MedChem. All drugs were diluted in DMSO. For in vitro experiments, cells were seeded at 300,000 per well in 6-well plates and treated with 2 doses of inhibitor 3 days apart. Cells were collected after 6 days after the first dose for flow cytometric analysis or fixed for 2 hours in formalin and spun down on slides for immunofluorescence analysis. To assess PSMA cell surface expression by flow cytometry, cells were dissociated, washed once with FACS buffer (PBS + 10%FBS), and stained with PE anti–human PSMA antibody (BioLegend, 342504) by resuspending cells in 100 μL of FACS buffer and in 5 μL of PE anti-PSMA antibody and by incubating cells on ice in the dark for 20 minutes. Cells were washed 3 times with FACS buffer before analysis on a Sony SH800 cell sorter (Sony Biotechnology). All downstream analyses were performed using FlowJo (v10). For IHC studies, cells were collected by trypsinization, fixed for 2 hours in 10% buffered formalin, and used for cytospin preparation as described previously.

### In vivo experiments.

All surgical procedures were performed under isoflurane anesthesia. LuCaP 35CR tumors (1 mm^3^) were surgically implanted s.c. into castrated male CB17SCID mice (The Jackson Laboratory) (59). When tumors reached a volume between 150 mm^3^ and 200 mm^3^, mice were administered CUDC-907 (Curis Inc.), a combined pan-HDAC/PI3K inhibitor dissolved in 30% captisol (Thermo Fisher Scientific). Treated mice received 75 mg/kg/day for 5 days, followed by 2 days off treatment, repeated for a total of 21 days or until the diameter of the tumor reached 2 cm. The same volume of 30% captisol, in the same manner as described above, was used to treat the vehicle group. At the end of the experiment, mice were euthanized, and tumors were harvested and fixed in 10% formalin before IHC analyses.

### Human tissue samples.

Metastatic cancer samples were collected as part of the Prostate Cancer Donor Program at the UW, and tissue microarrays (TMA) containing 52 patient samples from available tissues specimens from different metastatic sites (median number of sites per patient, 7; range, 1–21) were constructed as described previously (Supplemental Table 2) (60). None of these patients received prior PSMA-directed therapies.

### IHC staining.

For chromogenic PSMA IHC staining, slides were deparaffinized and steamed for 45 minutes in Target Retrieval Solution (Agilent/Dako). The primary PSMA antibody (Agilent, M3620, clone 3E6) was used at 1:50 dilution. Immunocomplexes were detected using PV poly-HRP anti–mouse IgG (Leica Microsystems, PV6114) with DAB as the chromogen. For MUC1, CEACAM5, MSLN, and CDK6 staining, the following antibodies and pretreatment conditions were used: MUC1 (target retrieval solution, Agilent, M0613, clone E29, 1:20), CEACAM5 (target retrieval solution, Agilent, M7072, clone II-7, 1:20), MSLN (target retrieval solution, MilliporeSigma, 439R-1, 1:15), and CDK6 (antigen unmasking solution, Vector Labs, H-3300-250; Abcam, ab124821, 1:25). PV Poly-HRP anti–mouse IgG (Leica Microsystems, PV6114) and anti–rabbit IgG (Leica Microsystems, PV6119) were used as secondary antibodies. Immunocomplexes were detected using the Biotin XX Tyramide SuperBoost kit (Invitrogen, B40931) per manufacturer’s protocol with DAB as the chromogen. For AR and NE status assessment, antibodies specific to AR (Cell Signaling Technology, 5153T, 1:100), NKX3.1 (Thermo Fisher Scientific, 5082788, 1:50), synaptophysin (Thermo Fisher Scientific, RM9111S, 1:80), and INSM1 (Santa Cruz Biotechnology Inc., A-8, SC271408, 1:100) were used according to protocols described previously (61). For dual-immunofluorescence labeling of MUC1 and PSMA, a sequential staining protocol was used. MUC1 (Agilent, M0613) and PSMA (Agilent, M3620) were used at 1:20, followed by PV poly-HRP anti–mouse IgG (Leica Microsystems, PV6114). Target retrieval solution (Agilent Technologies, S169984-2) was used for antigen retrieval and antibody stripping. Immunocomplexes were detected using Tyramide Signal Amplification system from Thermo Fisher Scientific. For chromogenic IHC, tissue sections were counterstained with hematoxylin, and the slides were digitized on a Ventana DP 200 Slide Scanner (Roche). Membranous PSMA expression was scored in a blinded manner by 2 pathologists, whereby the optical density level (“0” for no brown color, “1” for faint and fine brown chromogen deposition, and “2” for prominent chromogen deposition) was multiplied by the percentage of cells at each staining level, resulting in a total score range of 0–200. The final score for each sample represents the average of 2 duplicate tissue cores. For immunofluorescence studies, Alexa Fluor 568 Tyramide (Invitrogen, B40956) was used for signal amplification. Slides were counterstained with DAPI (Thermo Fisher Scientific) and mounted with Prolong (Thermo Fisher Scientific), and fluorescence images were captured using a Nikon Eclipse E800 microscope (Nikon).

### In silico expression analysis.

RNA-Seq data of bulk flash-frozen tissues from the SU2C/Prostate Cancer Foundation, LuCaP PDXs, and UW-TAN mCRPC cohorts were processed as described previously (24, 26). All subsequent analyses were performed using R. Gene abundance was quantified using GenomicAlignments (62). Molecular subtype classification (AR/NE status) was performed as described previously (22). Tumors were assigned to PSMA-low and PSMA-high categories by dividing them into those with PSMA expression below and above the mean across tumors for each data set. The data sets were then reduced to only include AR^+^/NE^–^ tumors. Differential expression between PSMA-low versus PSMA-high groups was assessed using limma (63) and was filtered for a minimum expression level using the filterByExpr function with default parameters prior to testing and using the Benjamini-Hochberg FDR adjustment. We then filtered the results to genes with FDR ≤ 0.05 and absolute value fold change > 2 in at least 2 data sets. These were further refined to cell surface (33) and tier 1 druggable targets (32) with the same thresholds in at least 1 data set. Genome-wide gene expression results were ranked by their limma statistics and used to conduct GSEA (64) to determine patterns of pathway activity utilizing the curated pathways from MSigDBv7.4. Single-sample enrichment scores were calculated using GSVA with default parameters using genome-wide log_2_ FPKM values as input, the 10-gene androgen-regulated signature (23) and 31-gene cell cycle proliferation (CCP) scores (29). Immune decomposition was performed using CIBERSORTx (30) with LM22 cell type signatures, B-mode batch correction, absolute mode, and 1,000 permutations. Tumors were assigned to PAM50 categories using a previously described classification method (31). We restricted the classification to luminal A, luminal B, and basal, removing Her2 and normal samples from the training set and centroid scores prior to classification. Groups displayed in box plots were compared using 2-sided Wilcoxon rank tests with Benjamini-Hochberg multiple-testing correction.

### DNA methylation analyses.

For WGBS analyses, DNA was extracted from LuCaP PDX lines (e.g., 77, 78, and 93), subjected to bisulfite conversion, and sequenced on an Illumina HiSeq 2500 instrument (Illumina) to an average coverage of 30***×***. Raw WGBS reads were first trimmed using Trim Galore (0.6.6) and then aligned to UCSC hg19 reference genome using Bismark (0.23.0) (65). Bismark was further used to deduplicate the alignments and extract methylation call files, which report the percentage of methylated cytosines and the coverage at each position. WGBS data are publicly available on Gene Expression Omnibus (GEO; accession no. GSE205056). A previously validated assay combining methylated-DNA precipitation and methylation-sensitive restriction enzyme digestion (COMPARE-MS) was used for site-specific DNA methylation analyses (66). In brief, DNA samples were digested with AluI and HhaI (New England Biolabs), and methylated DNA fragments were enrichment using recombinant MBD2-MBD (Clontech) immobilized on magnetic Tylon beads (Clontech). The precipitated DNA containing methylated DNA fragments was eluted and subjected to quantitative PCR (qPCR) using IQ SYBR Green Supermix (Bio-Rad) with primers specific to the second intron of *FOLH1* (hg19 chr11:49228686-49228864) forward (F): 5′- ACCACACTGAGGACGAGATG -3′ ; reverse (R): 5′- ATTGCCCTCACTCTCATCCC -3′. For quantitative assessment of locus-specific methylation levels, Ct values of the samples of interest were normalized to Ct values of the positive control (in vitro fully methylated male genomic DNA), and methylation indices were calculated (range, 0%–100%).

### ChIP.

ChIP experiments were performed following previously established protocols (67, 68) using anti-RNA polymerase II CTD repeat YSPTSPS phospho-serine 5 (Abcam, ab5408) and anti–Histone H3 (acetyl K27; Abcam, ab4729) antibodies. Recovered DNA was analyzed by qPCR using IQ SYBR Green Supermix (Bio-Rad) with primers specific to the transcriptional start site of *FOLH1* (hg19 chr11:49229852-49229951) F: 5′- AAGCCGAGGAGAAAGAAGCC -3′; R: 5′- TCCTTCACGAAACCGACTCG -3′. For in silico analyses of AR and H3K27ac ChIP-Seq studies, previously published data sets were accessed (38, 42, 43).

### Statistics.

Distributions of H-scores across molecular subgroups, defined by AR signaling and NE marker expression (22, 23), were compared using the Kruskal-Wallis test. Associations between H-scores and *FOLH1* mRNA levels in LuCaP PDX and UW-TAN samples were evaluated using linear regression. Heterogeneity of subgroups across metastatic sites in a given patient was quantified using hypergeometric probabilities that a randomly chosen pair of sites have different subgroups (69). Alternative measures of heterogeneity and Shannon and Simpson indices were obtained using the R package Vegan. Differences in distributions of heterogeneity scores in the UW-TAN for patients that died in 2003–2010 versus 2011–2019 were visualized using kernel density estimation and compared using Kolmogorov-Smirnov tests. Heterogeneity across metastatic sites in a given patient (intertumoral heterogeneity) and within a metastatic site (intratumoral heterogeneity) was summarized across patients using the mean outcome of 1,000 randomly sampled pairs and bias-corrected and accelerated 95% CI limits from the R package Bootstrap (25). In all analyses, *P* < 0.05 was considered statistically significant.

### Study approval.

In vivo studies were approved by the FHCC IACUC (IR 51048) and were performed in strict accordance with the guidelines in the Guide for the Care and Use of Laboratory Animals of the National Institutes of Health. Human tissue studies were approved by the IRB of the UW (protocol no. 2341). All rapid autopsy tissues were collected from patients who provided written informed consent under the aegis of the Prostate Cancer Donor Program at the UW.

## Author contributions

Research studies were designed by ES, CM, EC, RG, MPR, MTS, NHB, JKL, PSN, and MCH. Methodology was developed by ES, RAP, IMC, PM, BH, MPR, JYL, PSN, RG, and MCH. Experiments were conducted by ES, MPR, RAP, PM, EC, LA, AZ, IMC, JYL, BH, FYF, DAQ, LDT, JKL, CM, PSN, and MCH. Data were acquired and analyzed by ES, RG, MR, AZ, EC, RAP, NHB, MTS, JEH, IMC, BH, LDT, CM, PSN, and MCH.The manuscript was written by all authors. Reagents and data were provided by ES, MPR, RAP, IMC, BH, LDT, CM, FYF, DAQ, PSN, and MCH. VB contributed to methodology development and experiments performed. MA contributed to experiments performed. HB contributed to experiments performed. LK provided reagents data.

## Supplementary Material

Supplemental data

Supplemental table 1

Supplemental table 2

Supplemental table 3

Supplemental table 4

Supplemental table 5

Supplemental table 6

Supplemental table 7

Supplemental table 8

## Version 1 02/23/2023

In-Press Preview

## Version 2 04/10/2023

Electronic publication
