# Supplementary material for: Reversible epigenetic alterations mediate PSMA expression heterogeneity in advanced metastatic prostate cancer
Source: JCI Insight. 2023 Apr 10;8(7):e162907. doi: 10.1172/jci.insight.162907 (PMC10132157; doi:10.1172/jci.insight.162907)
Supplement: Supplemental data [file jciinsight-8-162907-s255.pdf]

SUPPPLEMENTARY DATA

PSMA expression heterogeneity in advanced metastatic prostate cancer is associated with reversible epigenetic alterations.

Erolcan Sayar, Radhika A. Patel, Ilsa Coleman, Martine P. Roudier, Ailin Zhang, Pallabi Mustafi, Jin-Yih Low, Brian Hanratty, Lisa Ang, Vipul Bhatia, Mohamed Adil, Hasim Bakbak, David A. Quigley, Michael T. Schweizer, Jessica E. Hawley, Lori Kollath, Lawrence D. True, Felix Y. Feng, Neil H. Bander, Eva Corey, John K. Lee, Colm Morrissey, Roman Gulati, Peter S. Nelson, Michael C. Haffner

Supplementary Figure 1. *Sayar et al.*

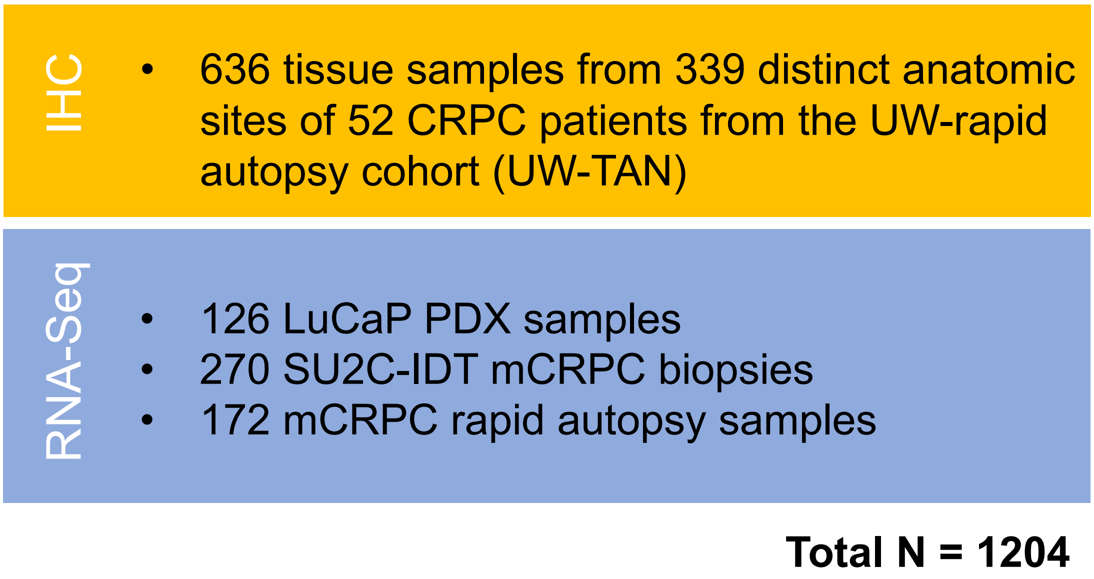

SUPPLEMENTARY FIGURE 1. Overview of samples included in the study.

Supplementary Figure 2. *Sayar et al.*

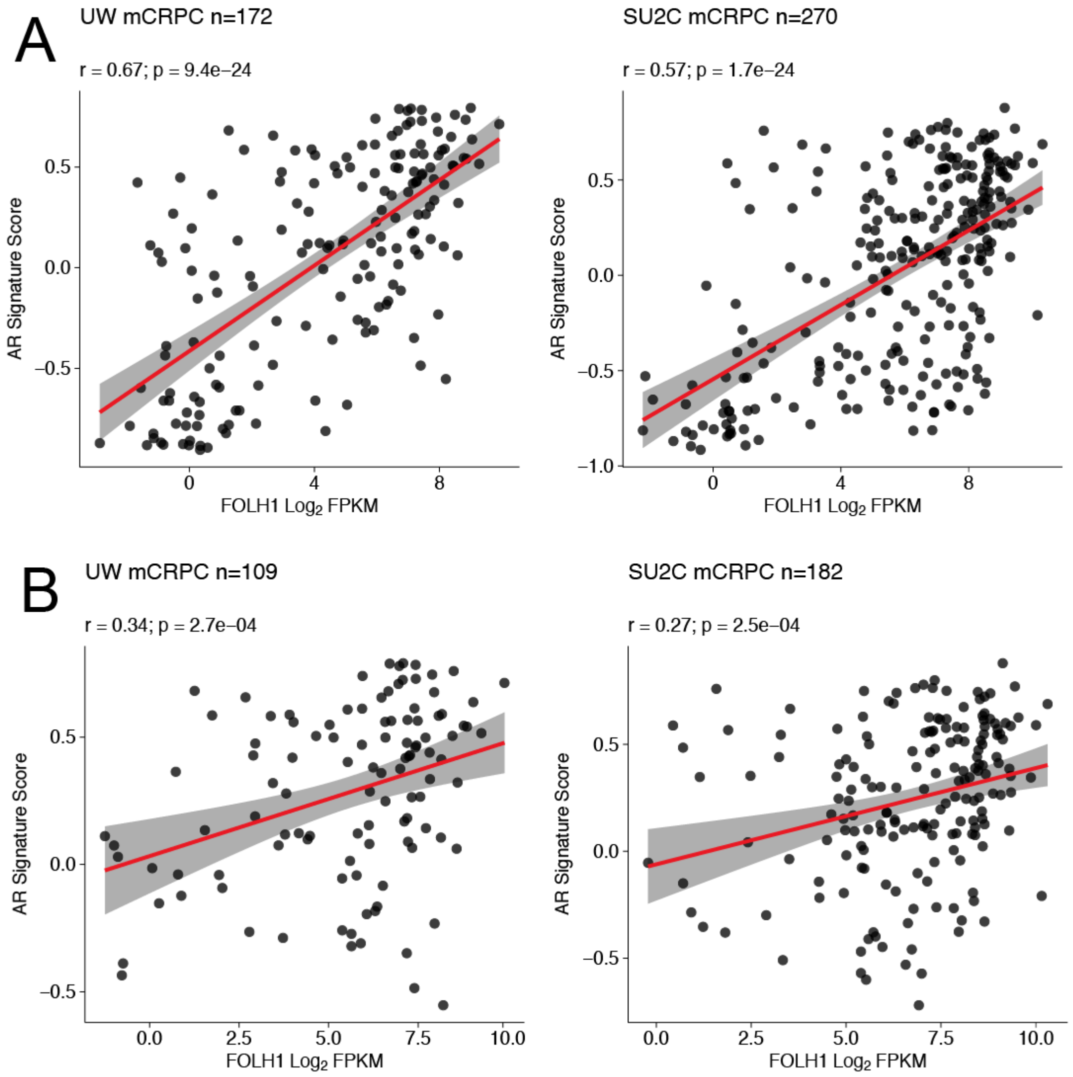

SUPPLEMENTARY FIGURE 2. Correlation plots show AR activity signature (AR10) scores and FOLH1 mRNA levels in **A.** all cases of the UW-TAN and SU2C cohorts and **B.** only AR+/NE- cases.

Supplementary Figure 3. *Sayar et al.*

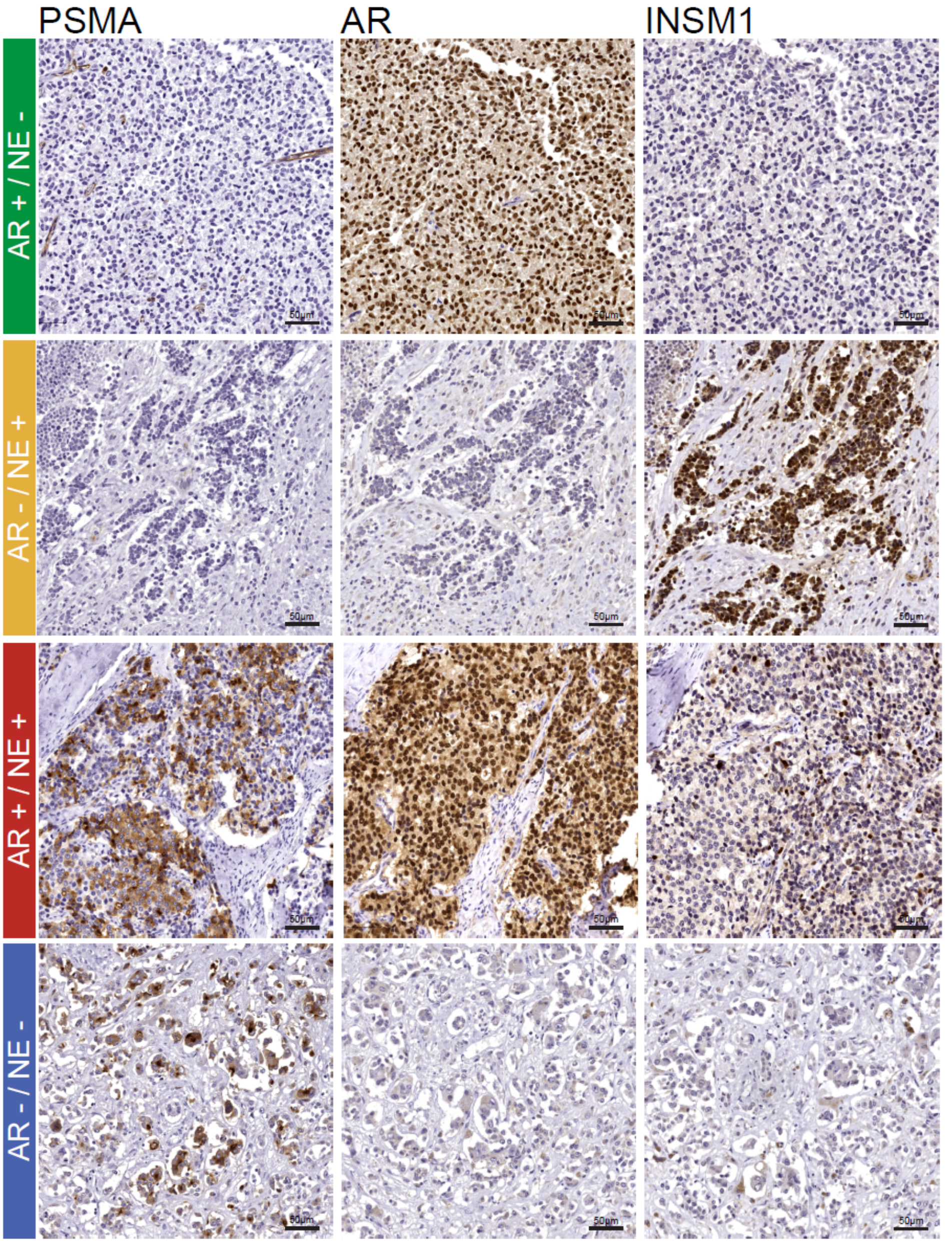

SUPPLEMENTARY FIGURE 3. Representative micrographs showing PSMA expression across different molecular subtypes. Absence of PSMA expression was seen in a subset of AR+/NE- cases. AR-/NE+ cases were uniformly negative for PSMA protein expression. In AR+/NE+ cases, PSMA levels were high, whereas AR-/NE- cases showed variable PSMA expression. Scale bars denote 50 μm.

Supplementary Figure 4. *Sayar et al.*

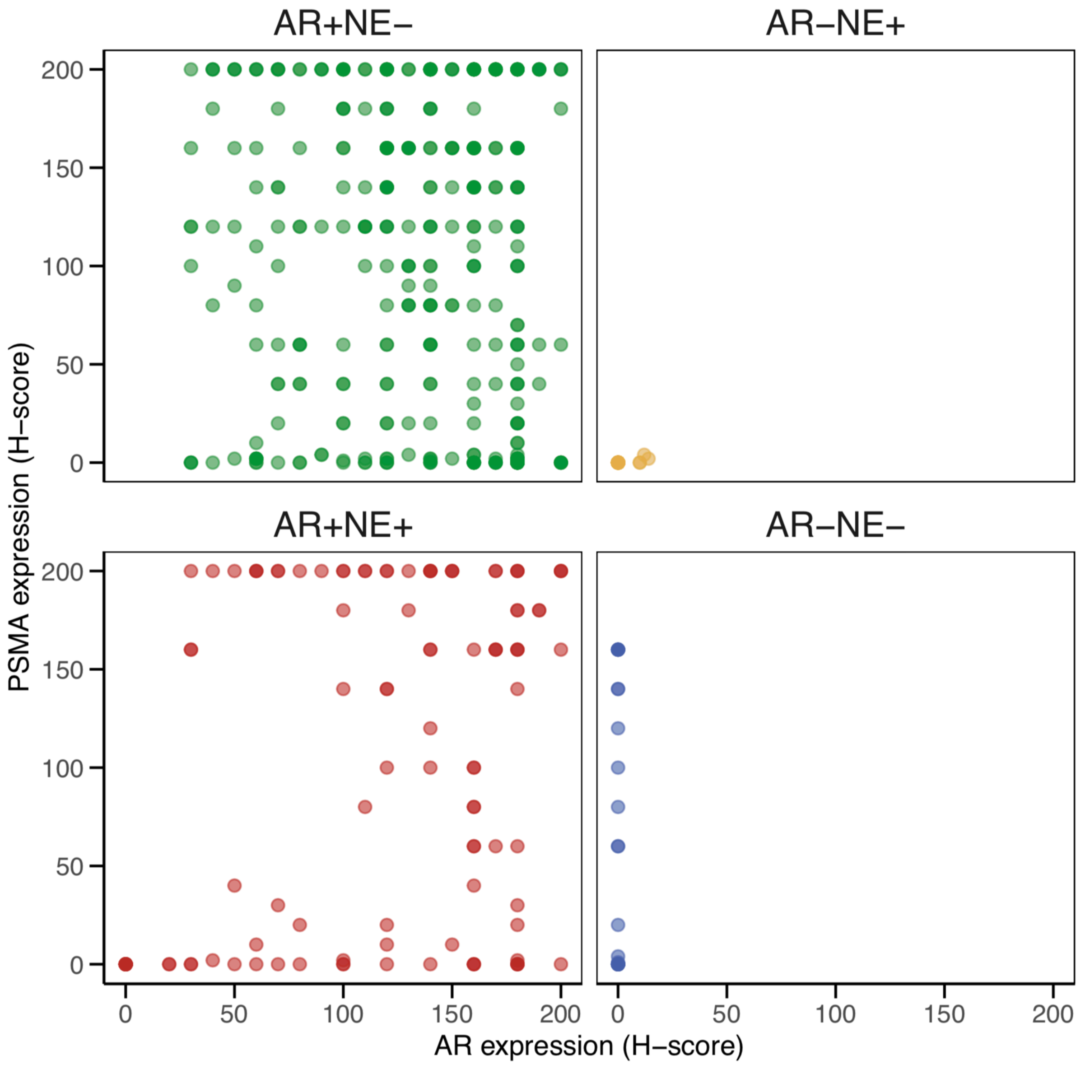

SUPPLEMENTARY FIGURE 4. Correlation plots show AR and PSMA protein levels (H-score) across different molecular subtypes. AR+ subtypes were defined as positivity for AR or NKX3.1 > H-score 20.

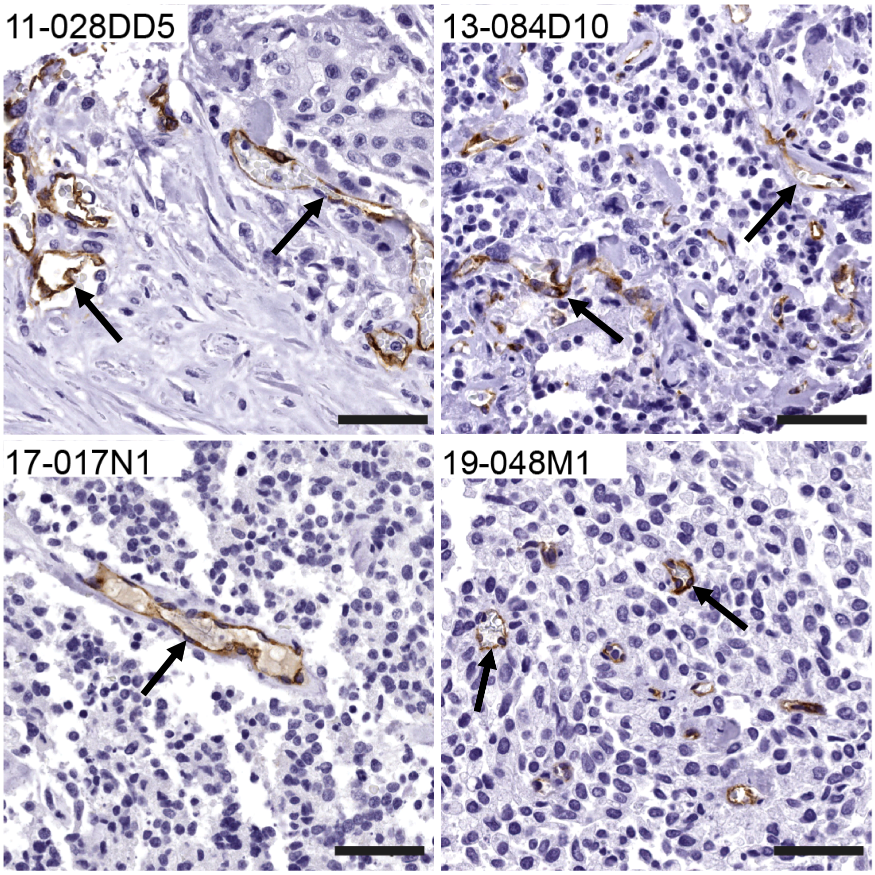

SUPPLEMENTARY FIGURE 5. Representative micrographs of PSMA negative tumors with PSMA expression in the tumor associated vasculature. Arrows show PSMA positive endothelial cells. Scale bars denotes 50 μm.

Supplementary Figure 6. *Sayar et al.*

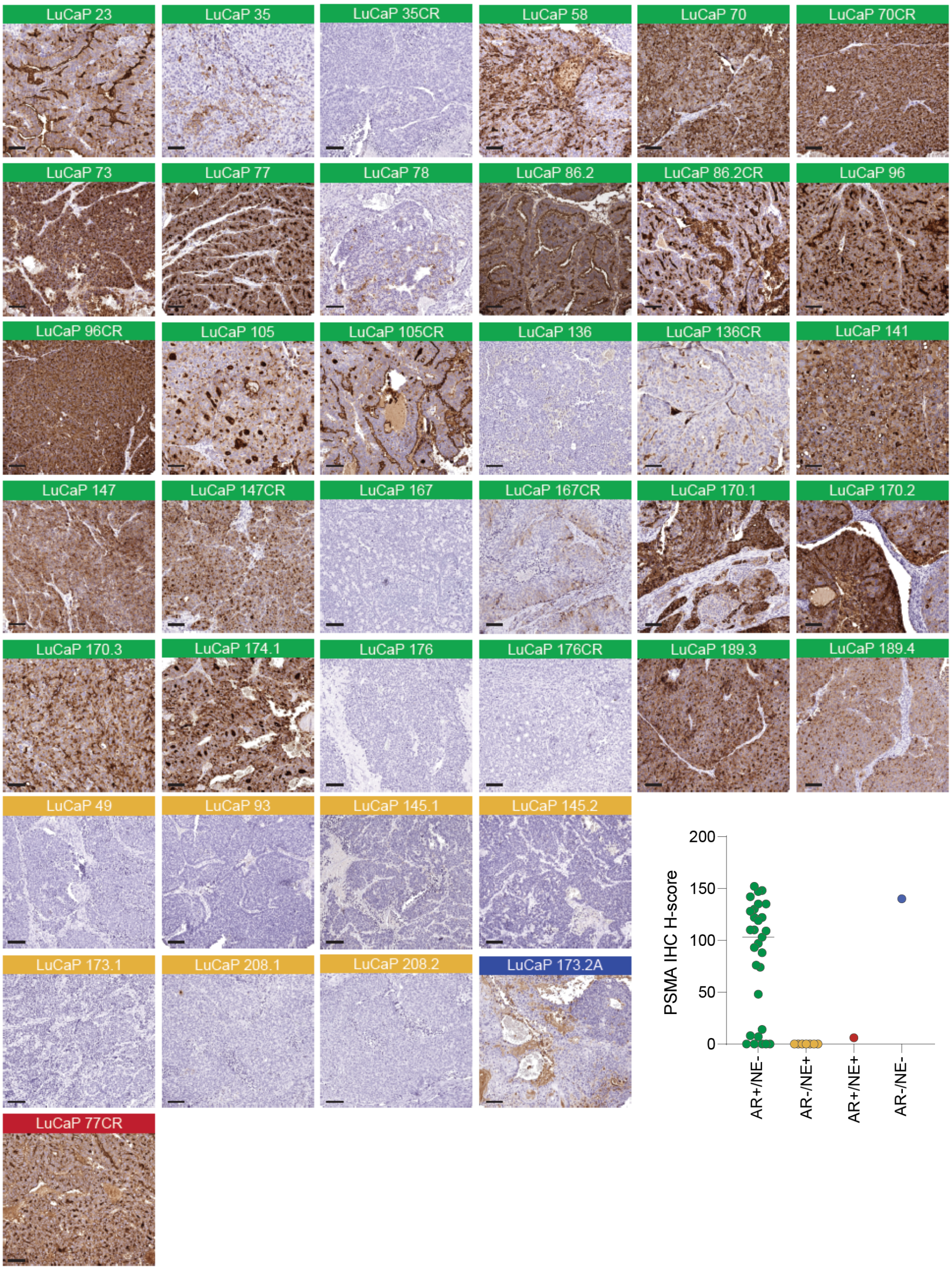

SUPPLEMENTARY FIGURE 6. PSMA protein expression as determined by immunohistochemistry in LuCaP PDX lines. Molecular phenotypes are color-coded as follows, AR+/NE-, Green; AR-/NE+, Yellow; AR+/NE+, Red; AR-/NE-, Blue. Bar graph show PSMA H-score distribution in LuCaP PDX lines across molecular subtypes. Scale bar denotes 100 μm.

Supplementary Figure 7. *Sayar et al.*

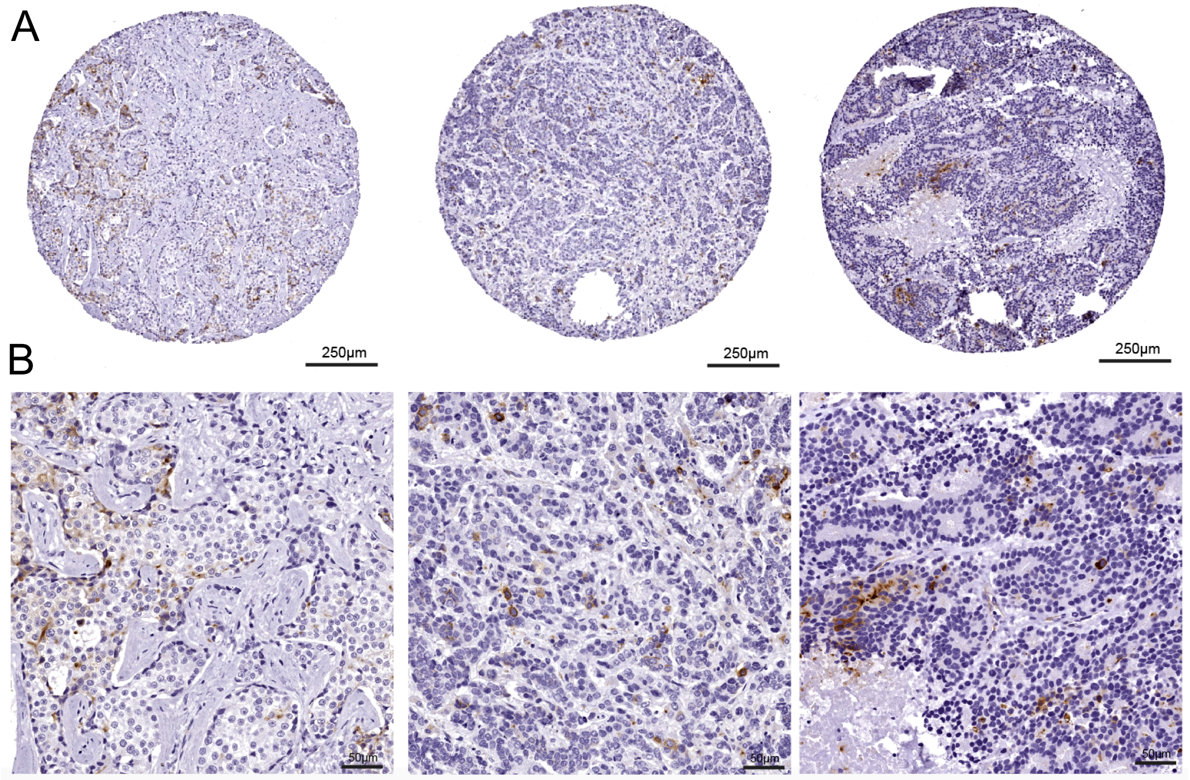

SUPPLEMENTARY FIGURE 7. Representative micrographs of cases with an H-score of 20. **A.** Low power overview of entire tissue cores shows focal patchy reactivity. **B.** Higher magnification images of areas with highest expression. Scale bars denotes 50 μm.

Supplementary Figure 8. *Sayar et al.*

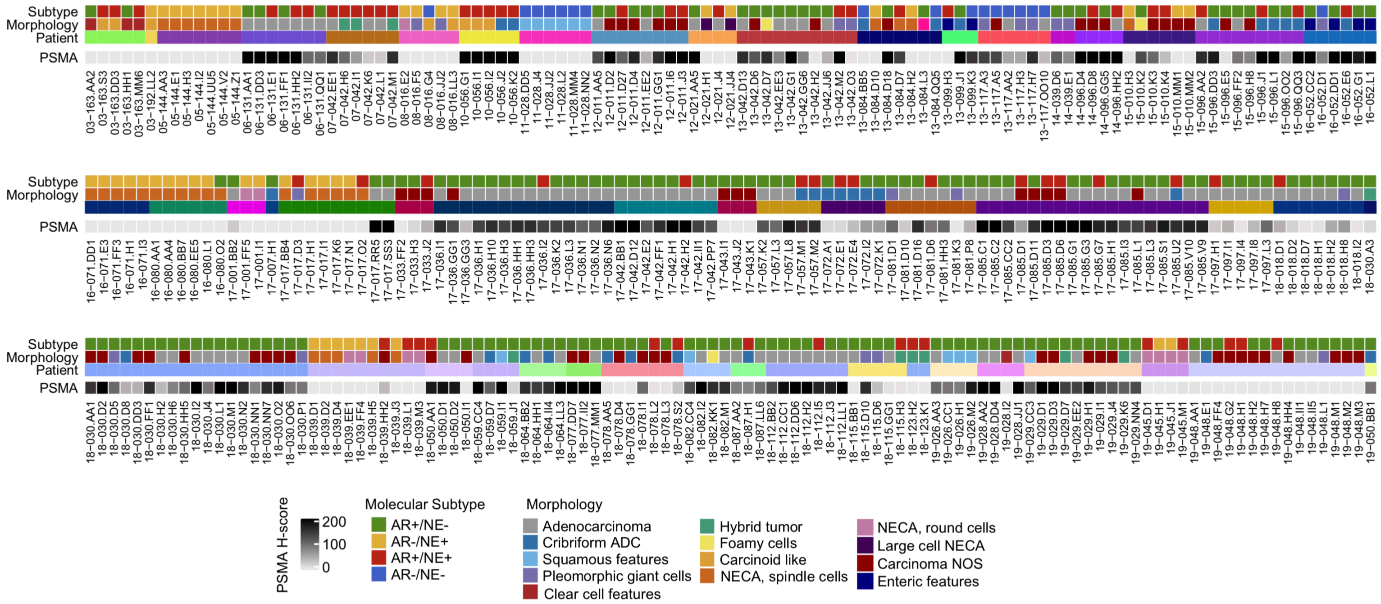

SUPPLEMENTARY FIGURE 8. Heat map showing molecular subclass, morphology and PSMA expression (H-score) in 52 patients included in this study. Note that H-scores shown here represent averaged values of all cores for a given anatomic site.

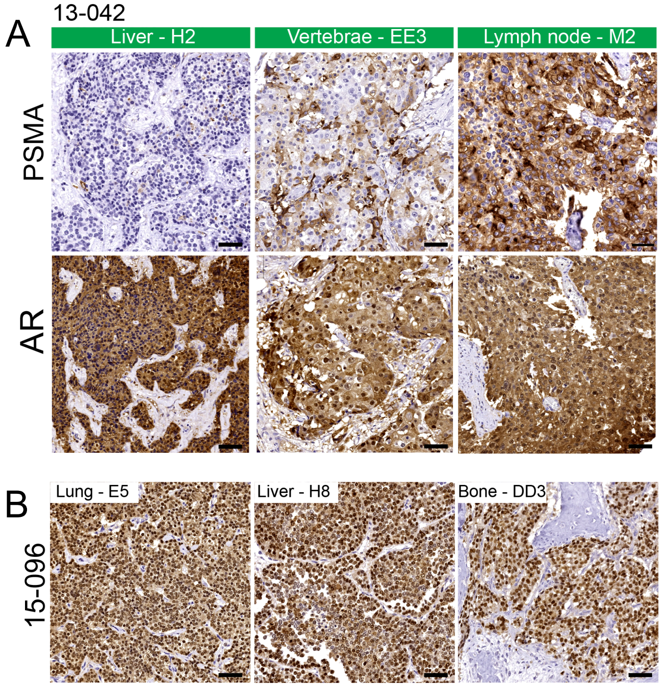

SUPPLEMENTARY FIGURE 9. A. Heterogeneous PSMA expression between different metastatic sites in case 13-042. Note that despite absence of PSMA expression, AR is expressed at high levels in all sites. B. Micrographs of AR IHC stains corresponding to PSMA IHCs shown in Figure 2C show robust AR expression in all metastatic sites. Scale bars denote 50 μm.

Supplementary Figure 10. *Sayar et al.*

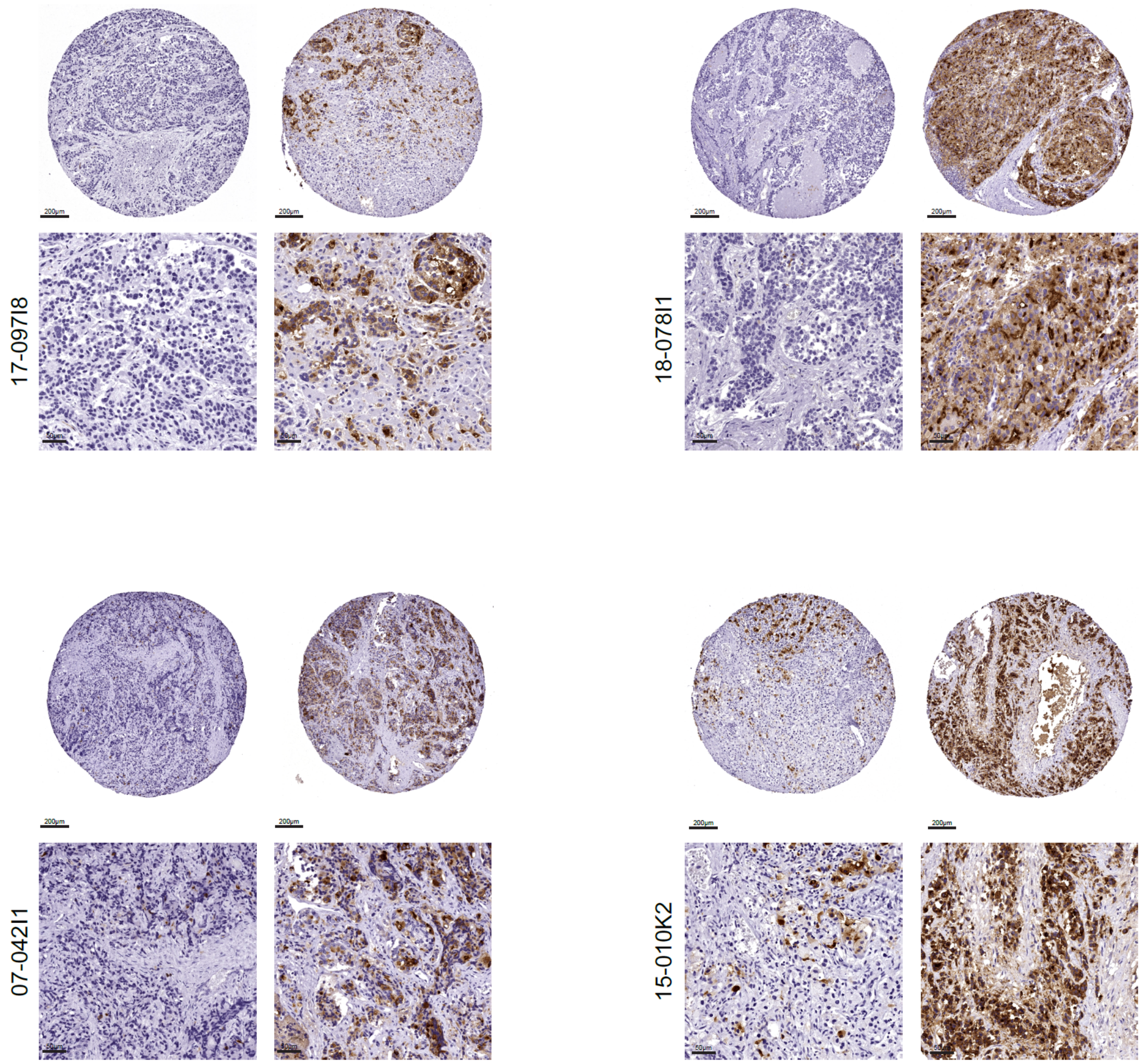

SUPPLEMENTARY FIGURE 10. Intra-tumoral heterogeneity of PSMA expression. Micrographs show 2 topographically separated tumor foci within a metastasis with striking differences in PSMA expression in 4 cases (17-097I8, 18-078I1, 07-042I1, 15-010K2). For each case, top panel shows entire TMA cores, bottom panel shows higher magnification view (20x).

Supplementary Figure 11. *Sayar et al.*

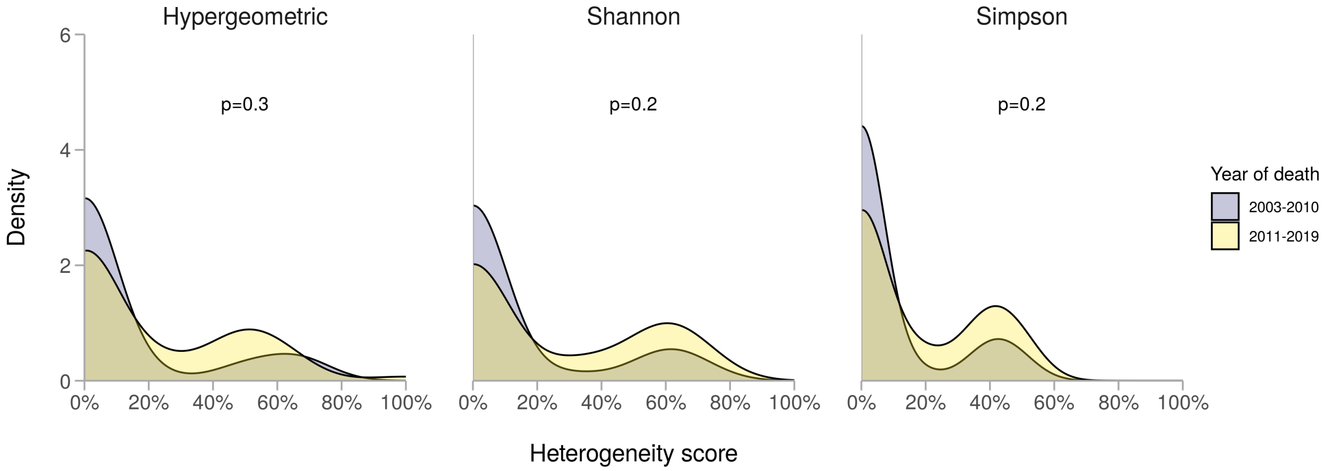

SUPPLEMENTARY FIGURE 11. Density plots show the distribution of heterogeneity scores (Hypergeometric, Shannon and Simpson) for cases from 2003-2010 (pre- abiraterone and enzalutamide) and 2011-2019 (abiraterone and enzalutamide era). In addition to the TMA set used throughout the manuscript, an additional smaller TMA set enriched for samples collected prior to 2010 was included in this analysis. In total, 31 cases of men who died between 2003 and 2010 (pre- abiraterone and enzalutamide) and 53 wo died between 2011 and 2019 (abiraterone and enzalutamide era) were included. Although, there was trend that patients who died between 2011-2019 appeared to have greater PSMA heterogeneity compared to patients who died in 2003-2010, the differences between subgroups were not statistically significant (P-values from 2-sided Kolmogorov-Smirnov).

Supplementary Figure 12. *Sayar et al.*

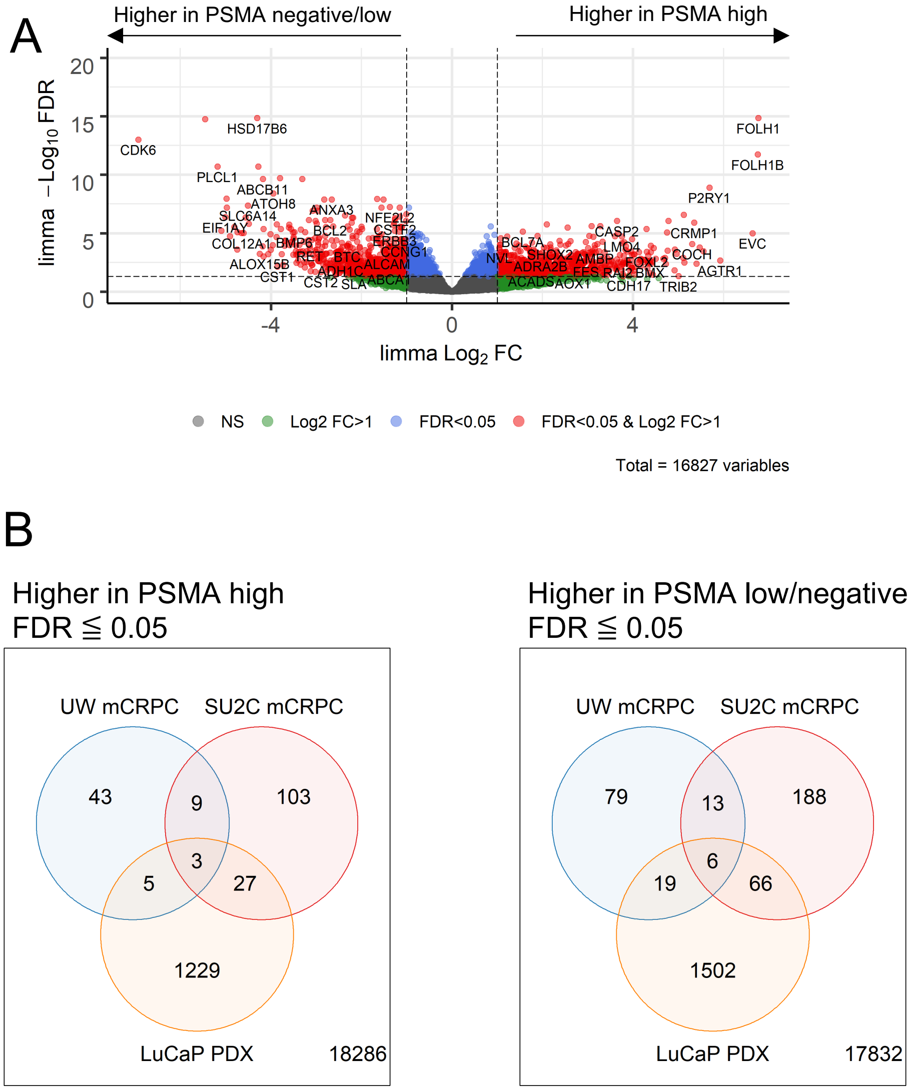

SUPPLEMENTARY FIGURE 12. A. Volcano plot depicting differentially expressed genes in PSMA negative/low and PSMA high AR+/NE- LuCaP PDX tumors. Note, that the high expression of CDK6 in tumors with low PSMA expression. **B.** Venn diagrams show the intersection of genes with increased expression in PSMA high and PSMA low tumors across 3 independent cohorts (SU2C-IDT, metastatic biopsies; UW-TAN, rapid autopsy; LuCaP PDX, patient derived xenografts) with a significance cutoff of FDR ≦ 0.05. For all analyses, only AR+/NE- tumors were included.

Supplementary Figure 13. *Sayar et al.*

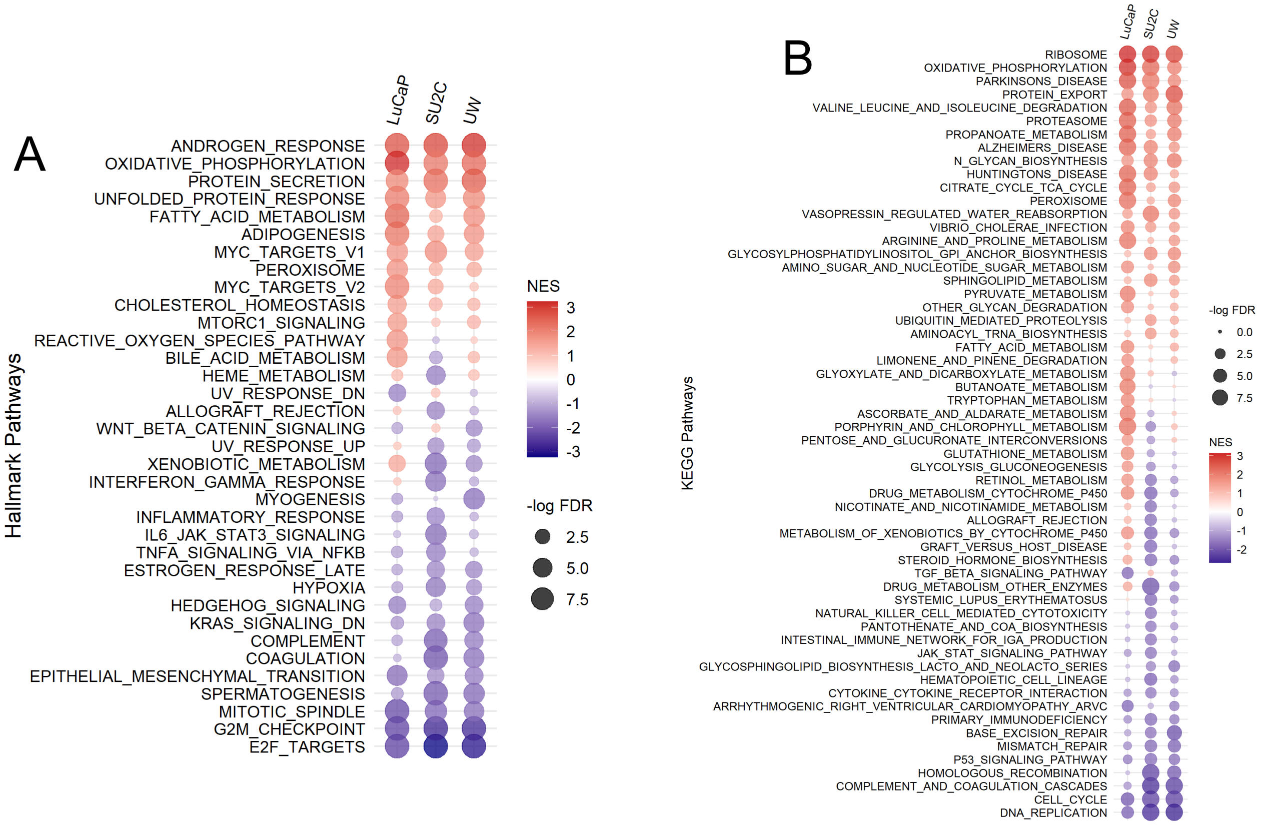

SUPPLEMENTARY FIGURE 13. Gene set enrichment analyses using **A.** Hallmark Pathways and **B.** KEGG Pathways show gene sets enriched in PSMA high (red) and PSMA (blue) low/negative tumors of all molecular subtypes (unselected cohorts).

Supplementary Figure 14. *Sayar et al.*

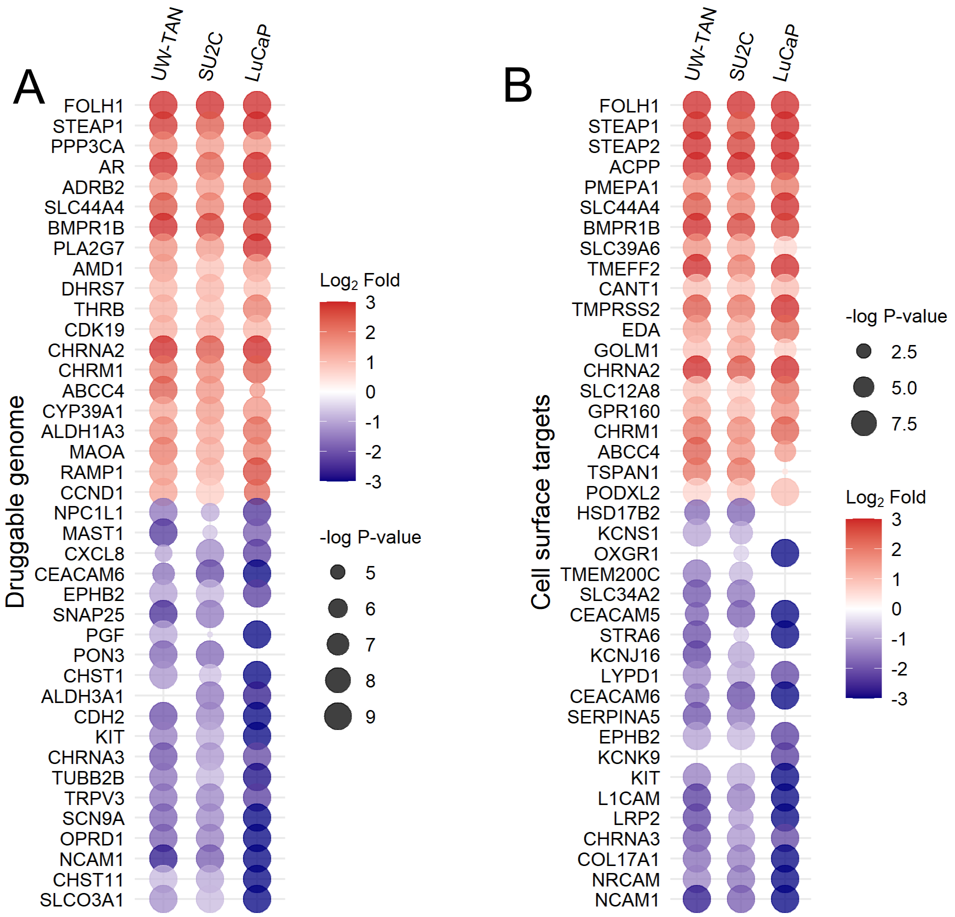

SUPPLEMENTARY FIGURE 14. Drug target analysis in unselected cohorts (all molecular subtypes) **A.** Heatmap of top 20 differentially expressed genes with annotated drug target properties from the druggable genome database (rank ordered based on fold expression difference) between PSMA high (red) and PSMA low/negative (blue) tumors in UW-TAN, SU2C and LuCaP PDX cohorts. **B.** Top 20 differentially expressed genes encoding for cell surface proteins between PSMA high (red) and PSMA low/negative (blue) tumors in UW-TAN, SU2C and LuCaP PDX. Heatmaps are sorted by rank order based on mean fold change differences and directionality is color coded, red, higher in PSMA high; blue, higher in PSMA low/negative.

Supplementary Figure 15. *Sayar et al.*

A PSMA_HIGH_UP_50: NES 2.72; FDR < 0.0001
PSMA_LOW_UP_50: NES -1.88; FDR = 0.002

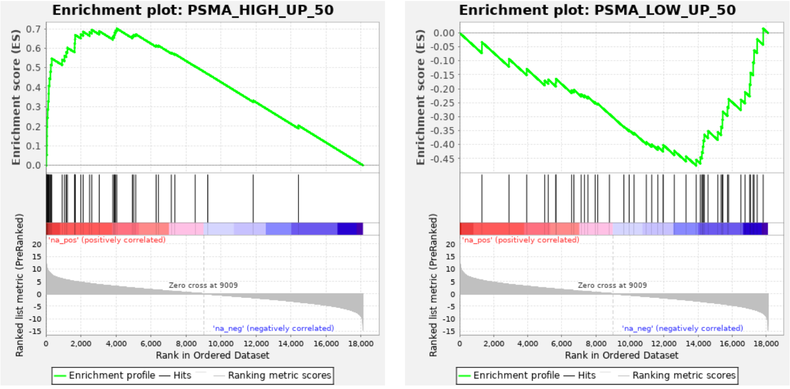

B PSMA_HIGH_UP_50: NES 2.81; FDR < 0.0001
PSMA_LOW_UP_50: NES -1.06; FDR = 0.368

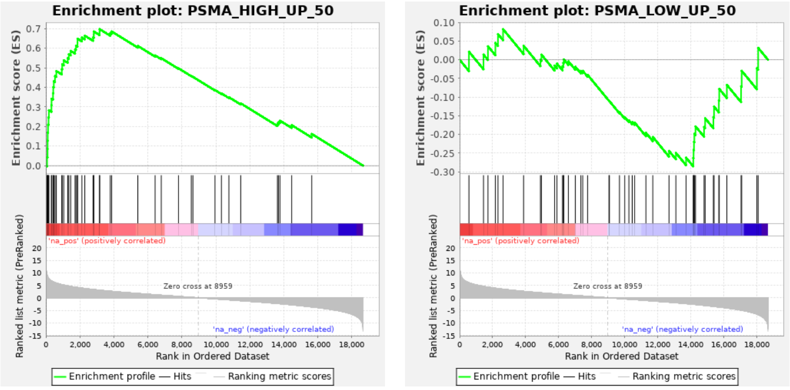

SUPPLEMENTARY FIGURE 15. Gene set enrichment analysis (GSEA) show enrichment of the PSMA_high_up gene set in AR+ tumors and the PSMA_low_up gene set in AR- tumors in **A.** SU2C (total N=270, AR+=205, AR-=65) and **B.** (total N=172, AR+=126, AR-=46).

Supplementary Figure 16. *Sayar et al.*

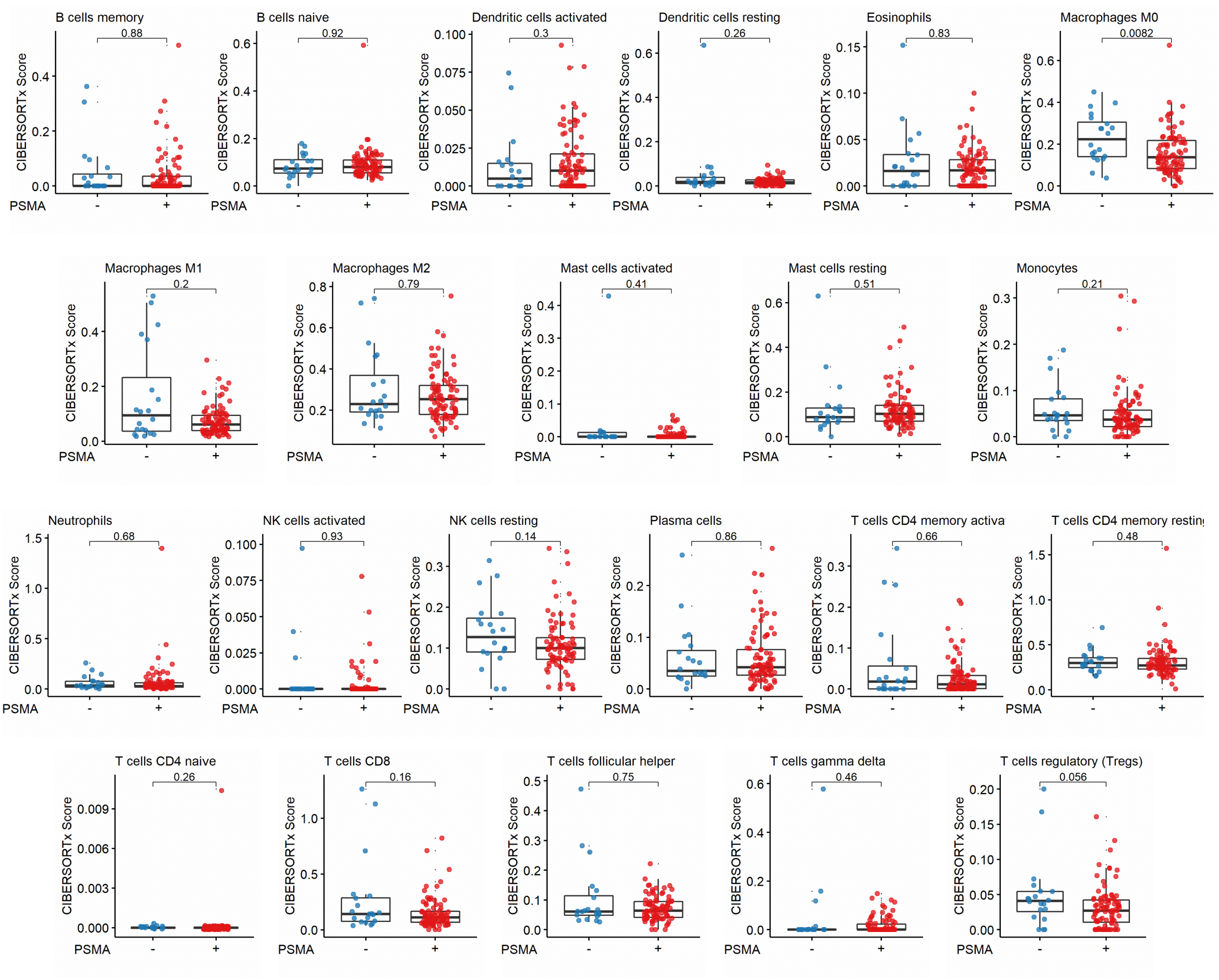

SUPPLEMENTARY FIGURE 16. CIBERSORT analysis of 109 metastatic CRPC samples (only AR+/NE-) from the UW-TAN rapid autopsy cohort. Note the statistically significant difference in M0 macrophages and T regulatory cells between PSMA high (red) and PSMA null/low tumors (blue).

Supplementary Figure 17. *Sayar et al.*

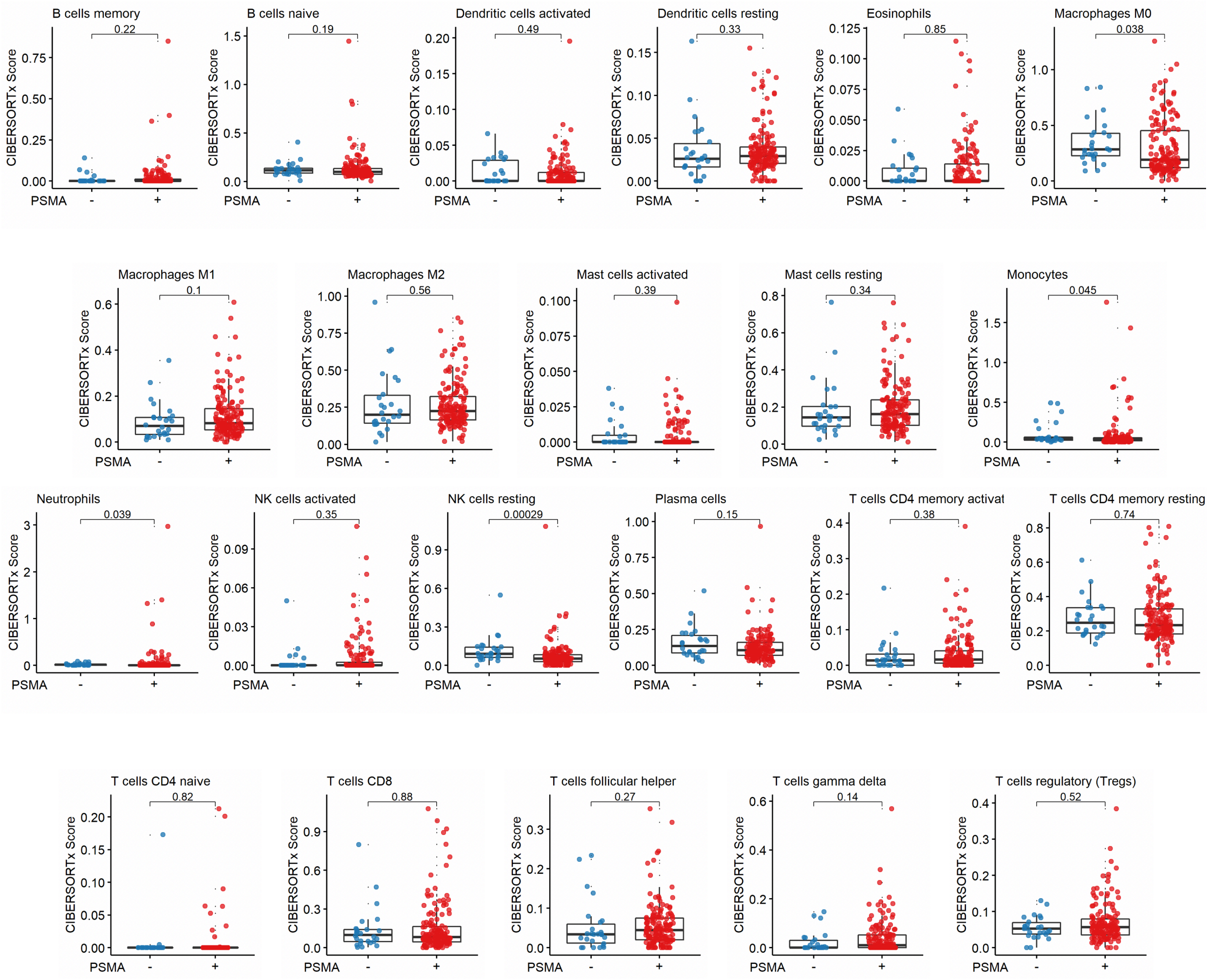

SUPPLEMENTARY FIGURE 17. CIBERSORT analysis of 182 metastatic CRPC samples (only AR+/NE-) for SU2C-IDT. Note the statistically significant difference in M0 macrophages, monocytes, neutrophils and NK cells (resting) between PSMA high (red) and PSMA null/low tumors (blue).

Supplementary Figure 18. *Sayar et al.*

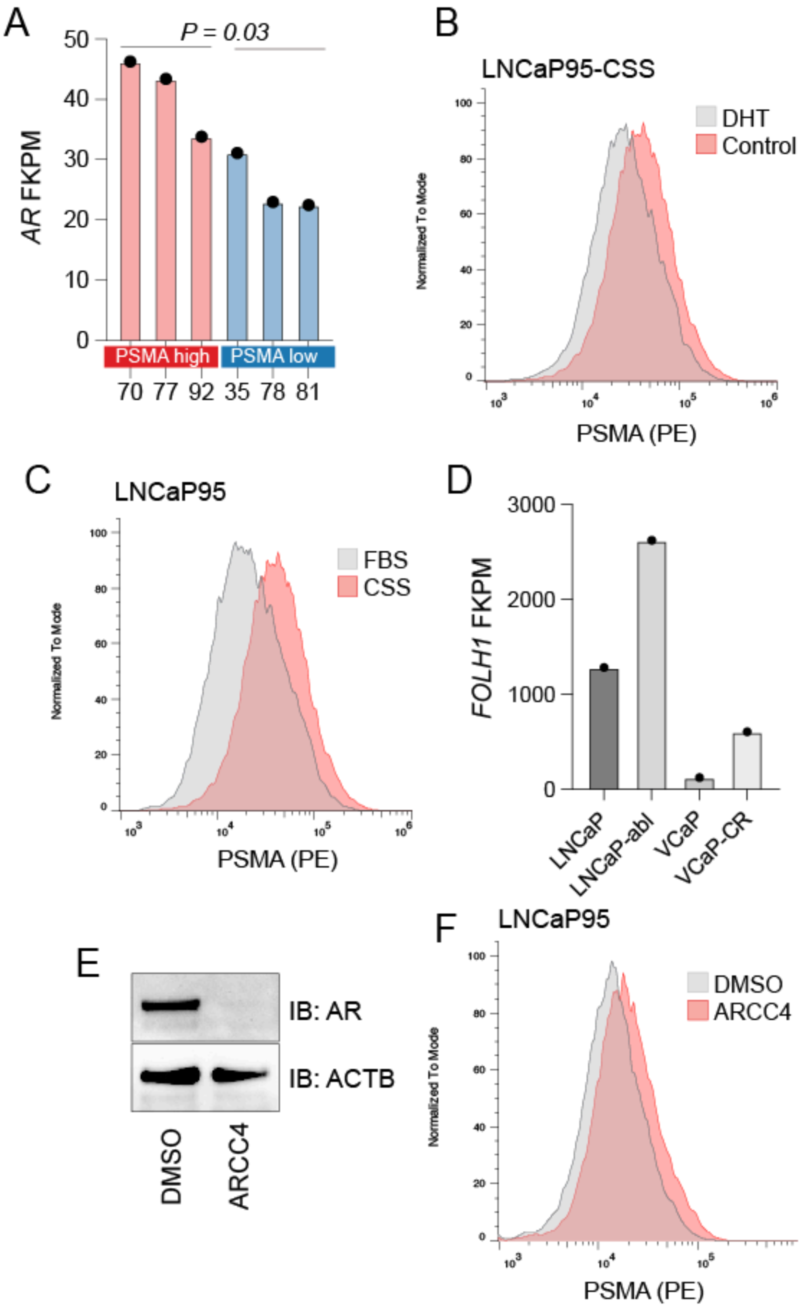

SUPPLEMENTARY FIGURE 18. A. AR mRNA expression in PDX tumors with high and low PSMA expression. Suppression of PSMA expression by **B.** DHT or **C.** growth in androgen containing media for 6 days (FBS, denotes fetal bovine serum, CSS, charcoal stripped serum) in LNCaP95 cells. **D.** FOLH1 mRNA expression in androgen dependent (LNCaP, VCaP) and androgen-independent (LNCaP-abl, VCaP-CR) cell lines. **E.** Western blot showing efficient AR depletion in LNCaP cells treated for 72h with 5μM ARCC-4. **F.** Corresponding flow cytometry plots showing increased PSMA expression in ARCC4 treated cells.

Supplementary Figure 19. *Sayar et al.*

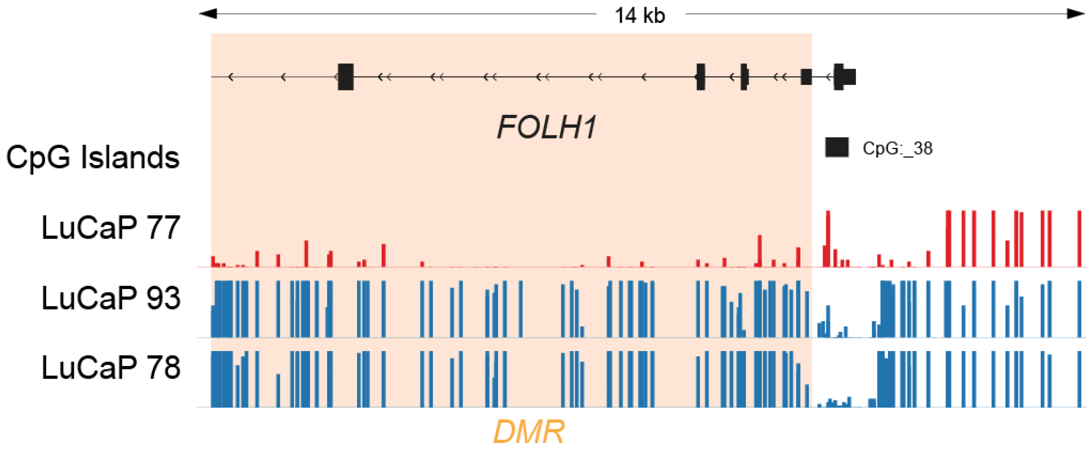

SUPPLEMENTARY FIGURE 19. IGV tracks of WGBS data of LuCaP 77 (PSMA high) and LuCaP 93 and 78 (PSMA negative) show gain of CpG methylation in the *FOLH1* gene body, but not the CpG island at the transcriptional start site in PSMA negative lines.

Supplementary Figure 20. *Sayar et al.*

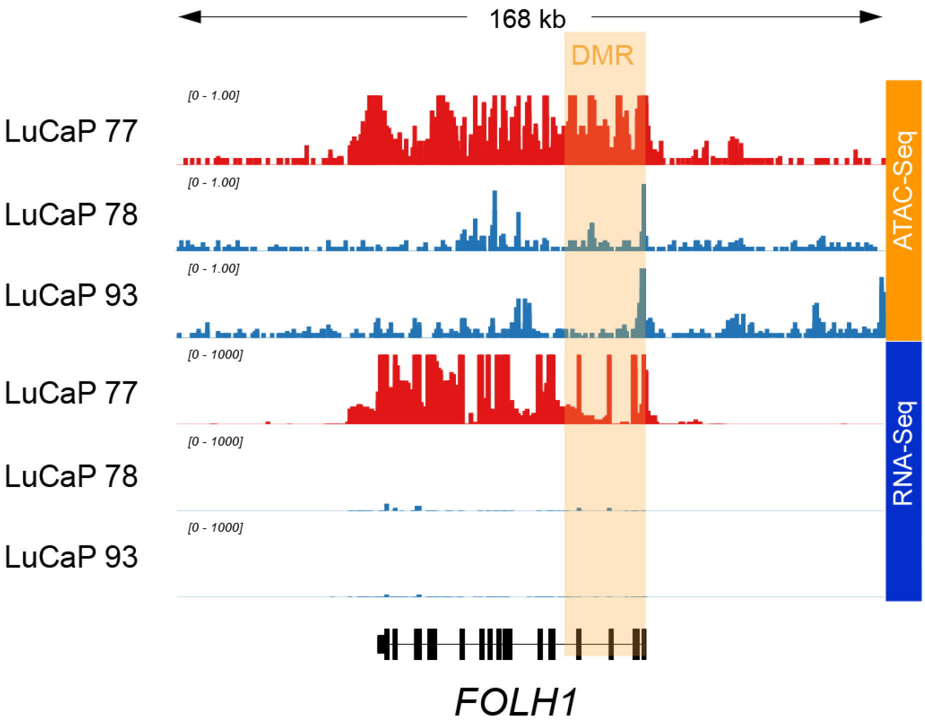

SUPPLEMENTARY FIGURE 20. Differences in chromatin accessibility, as determined by ATAC-seq and mRNA expression, as determined by RNA-seq of the *FOLH1* gene locus. The differentially methylated region (DMR) identified by DNA methylation analyses is highlighted in orange. ATAC-seq data were derived from Cejas P. et al, Nat Comm, 2021 and RNA-seq data were from Labreque MP et al., Cancer Res, 2021.

Supplementary Figure 21. *Sayar et al.*

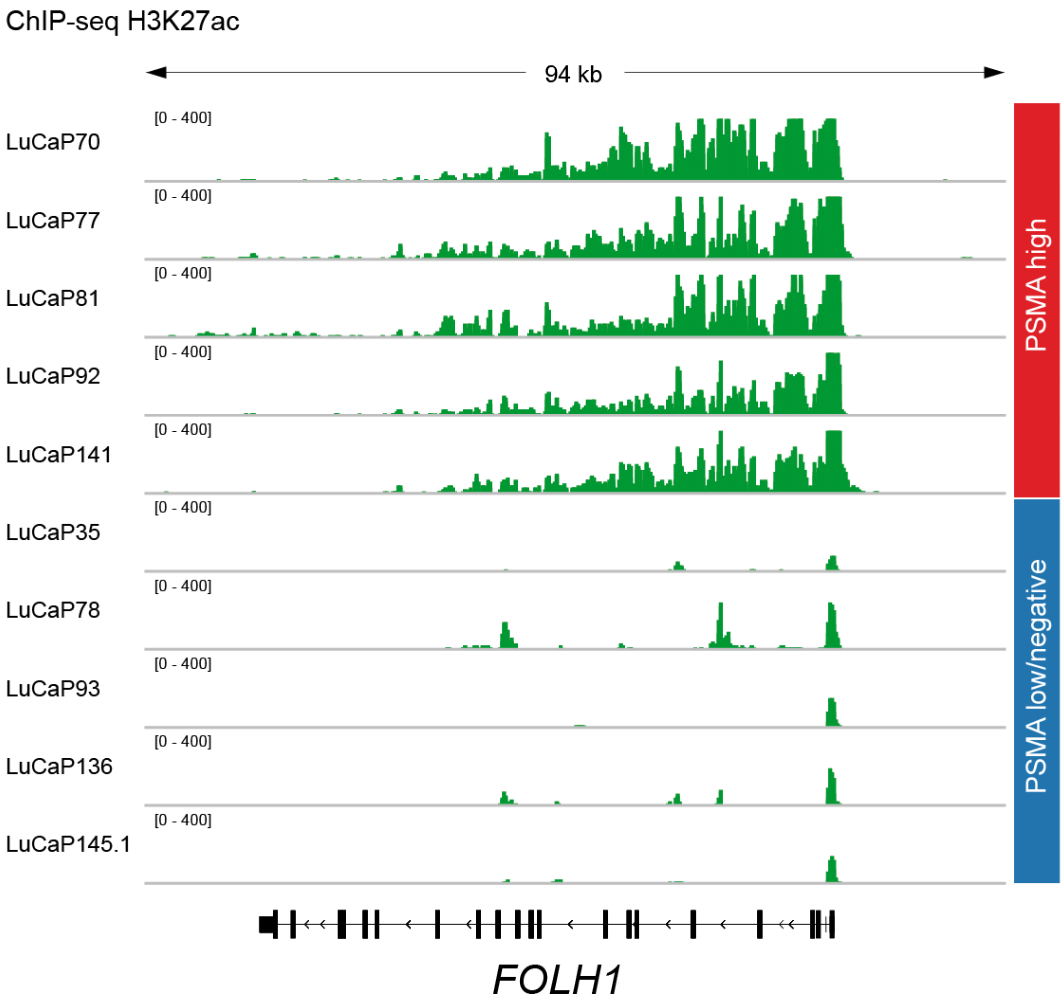

SUPPLEMENTARY FIGURE 21. LuCaP PDX models with high PSMA expression show strong enrichment for H3K27ac across the *FOLH1* gene body. This contrasts to tumors with low/negative PSMA expression which show only limited H3K27ac signals in this locus. H3K27ac ChIP-seq data were derived from Baca SC. et al, Nat Comm, 2021.

Supplementary Figure 22. *Sayar et al.*

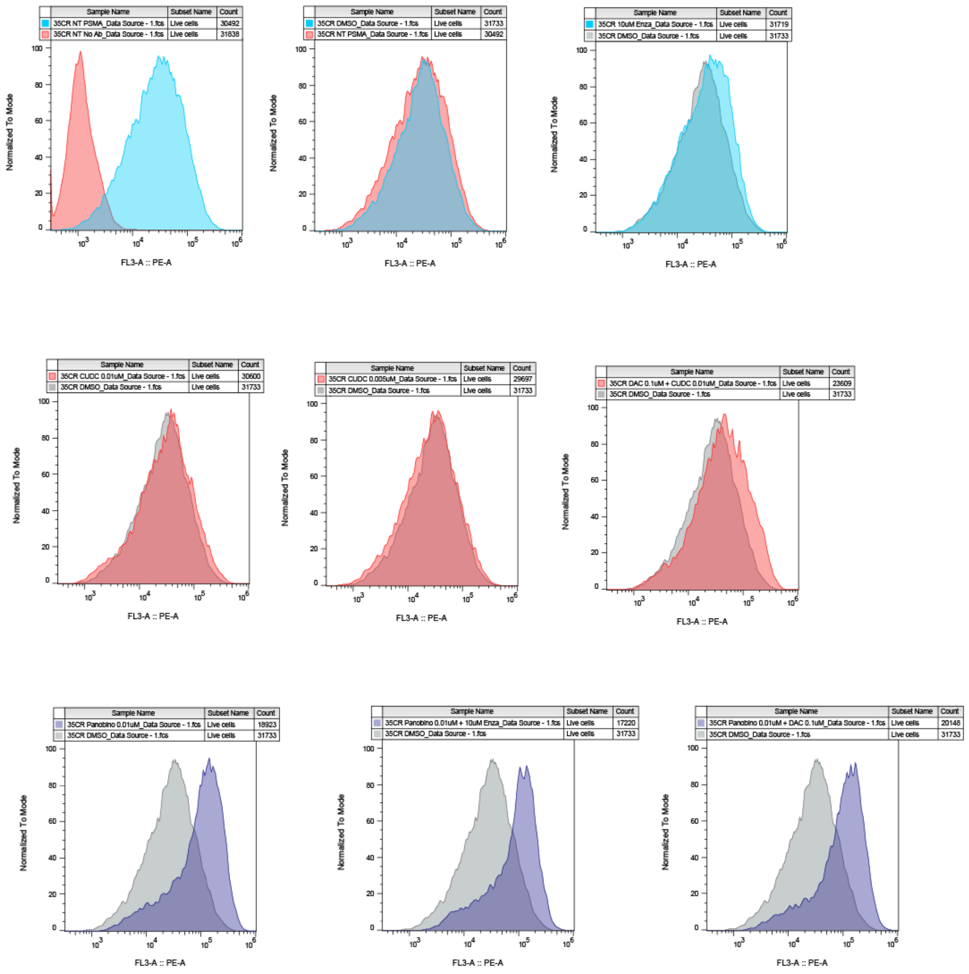

SUPPLEMENTARY FIGURE 22. Density plots show the distribution of PSMA labeling signals (x-axis) and their respective distributions in LuCaP 35 cells treated with solvent control (DMSO) or epigenetic modifiers (as indicated) for 6 days.

Supplementary Figure 23. *Sayar et al.*

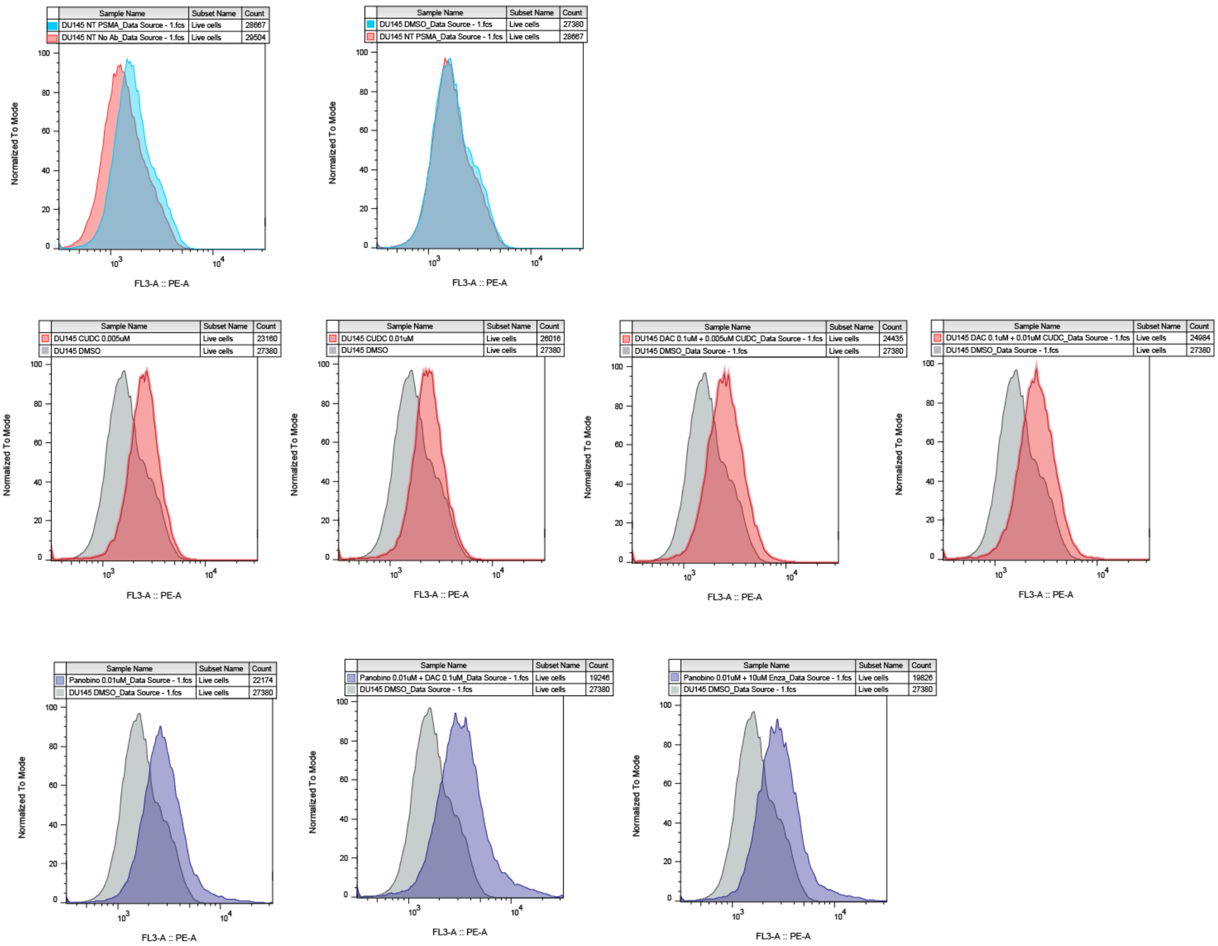

SUPPLEMENTARY FIGURE 23. Density plots show the distribution of PSMA labeling signals (x-axis) and their respective distributions in DU145 cells treated with solvent control (DMSO) or epigenetic modifiers (as indicated) for 6 days.

Supplementary Figure 24. *Sayar et al.*

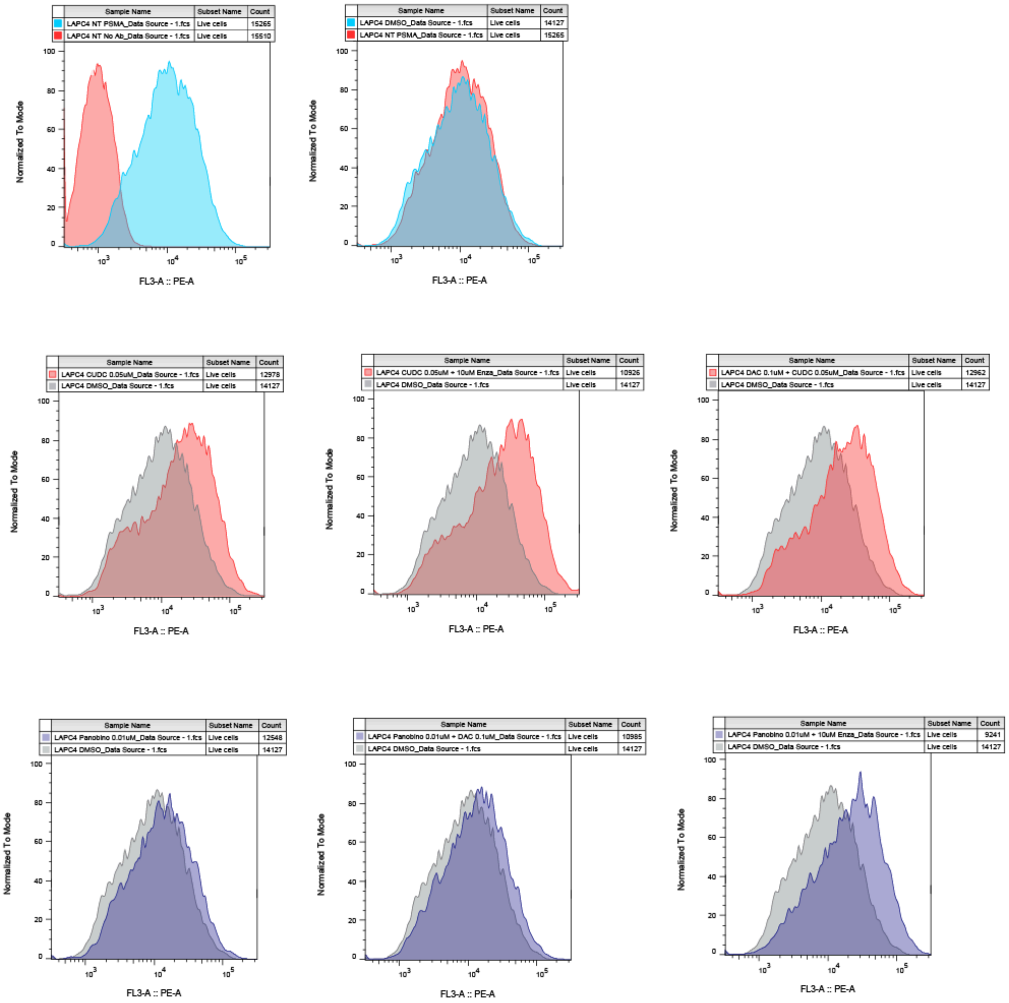

SUPPLEMENTARY FIGURE 24. Density plots show the distribution of PSMA labeling signals (x-axis) and their respective distributions in LAPC4 cells treated with solvent control (DMSO) or epigenetic modifiers (as indicated) for 6 days.

Supplementary Figure 25. *Sayar et al.*

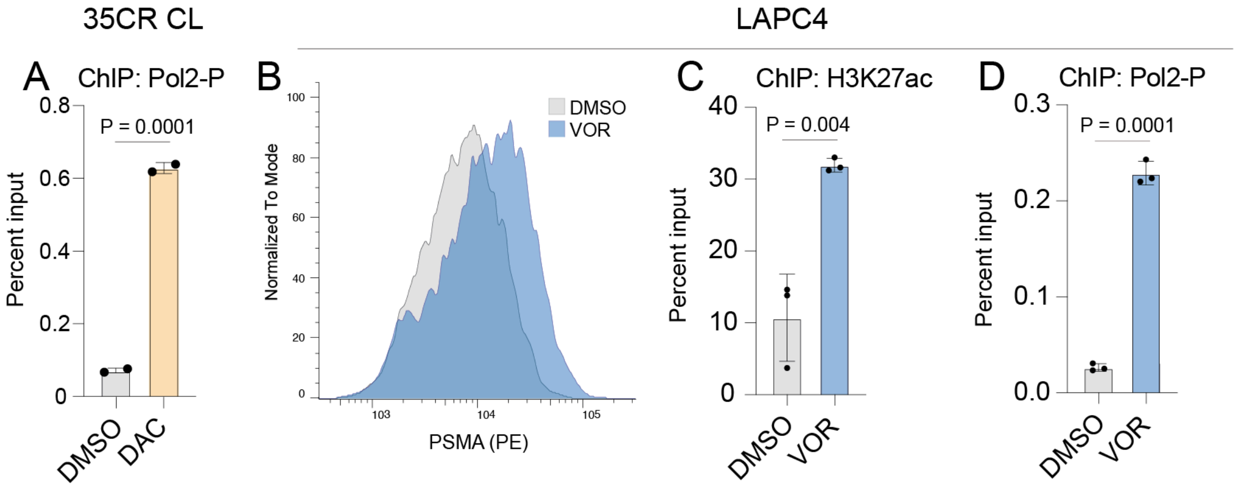

SUPPLEMENTARY FIGURE 25. A. Bar graph showing the enrichment of phosphorylated RNA polymerase 2 (Ser 5) by ChIP at the FOLH1 transcriptional start in 35CR CL cells treated with solvent control (DMSO) or decitabine (DAC) 500 nM for 6 days. Enrichment is normalized to input DNA. **B.** PSMA flow cytometry density plots show induction of PSMA expression upon treatment with the HDACi vorinostat (VOR, 6 days 1μM). ChIP-PCR assays confirm increased enrichment of **C.** H3K27ac and **D.** phosphorylated RNA polymerase 2 (Ser 5) in LAPC4 cells treated with 1μM VOR for 6 days compared to solvent (DMSO).
